# Supplementary material for: Risk factors for maternal mortality among 1.9 million women in nine empowered action group states in India: secondary analysis of Annual Health Survey data
Source: BMJ Open. 2020 Aug 20;10(8):e038910. doi: 10.1136/bmjopen-2020-038910 (PMC7440828; doi:10.1136/bmjopen-2020-038910)
Supplement: Supplementary data [file bmjopen-2020-038910supp001.pdf]

**Table-S1: Comparison of model estimates between unweighted and survey weighted data***

| Variables | Unweighted model | | Survey weighted model** | |
|---|---|---|---|---|
| | *OR* | *95% CI* | *OR* | *95% CI* |
| **Age** | | | | |
| 13-19 | 3.35 | (3.05, 3.68) | 3.66 | (3.27, 4.10) |
| 20-24 | 1.14 | (1.07, 1.22) | 1.16 | (1.07, 1.26) |
| 25-29 | 1.00 | -- | 1.00 | -- |
| 30-34 | 1.18 | (1.09, 1.28) | 1.19 | (1.08, 1.31) |
| 35-39 | 1.97 | (1.81, 2.15) | 1.92 | (1.72, 2.13) |
| 40-49 | 3.10 | (2.82, 3.41) | 2.80 | (2.49, 3.15) |
| **Distal factors** | | | | |
| **Place of residence** | | | | |
| Urban | 1.00 | -- | 1.00 | -- |
| Rural | 1.02 | (0.94, 1.11) | 0.97 | (0.88, 1.07) |
| **Religion** | | | | |
| Hindu | 1.00 | -- | 1.00 | -- |
| Buddhist | 0.83 | (0.21, 3.35) | 0.58 | (0.12, 2.77) |
| Christian | 0.73 | (0.60, 0.89) | 0.58 | (0.46, 0.74) |
| Jain | 1.10 | (0.41, 2.96) | 1.16 | (0.40, 3.36) |
| Muslim | 0.66 | (0.62, 0.72) | 0.64 | (0.59, 0.70) |
| Sikh | 1.13 | (0.66, 1.91) | 1.08 | (0.61, 1.92) |
| Other | 0.77 | (0.61, 0.97) | 0.72 | (0.54, 0.97) |
| **Social Group** | | | | |
| Other | 1.00 | -- | 1.00 | -- |
| Scheduled caste | 1.13 | (1.06, 1.20) | 1.10 | (1.02, 1.18) |
| Scheduled tribe | 1.52 | (1.42, 1.64) | 1.43 | (1.31, 1.56) |
| **Wealth index** | | | | |
| Wealthiest | 1.00 | -- | 1.00 | -- |
| Quintile 4 | 0.96 | (0.89, 1.04) | 0.99 | (0.89, 1.09) |
| Quintile 3 | 0.96 | (0.89, 1.04) | 0.97 | (0.88, 1.07) |
| Quintile 2 | 0.98 | (0.91, 1.06) | 0.97 | (0.89, 1.07) |
| Lowest quintile | 0.79 | (0.73, 0.85) | 0.81 | (0.74, 0.90) |
| **Has health scheme** | | | | |
| Yes | 1.00 | -- | 1.00 | -- |
| No | 2.78 | (2.52, 3.07) | 2.72 | (2.41, 3.07) |
| **Intermediate factors** | | | | |
| **Accessed a health facility** | | | | |
| Yes | 1.00 | -- | 1.00 | -- |
| No | 2.93 | (2.78, 3.09) | 2.93 | (2.75, 3.12) |
| **Proximal factors** | | | | |
| **Pregnancy complication or medical comorbidity** | | | | |
| No | 1.00 | -- | 1.00 | -- |
| Yes | 48.6 | (44.1, 53,7) | 50.2 | (44.5, 56.6) |

*Estimates shown are for the final logistic regression model with adjustment for all distal, intermediate and proximal factors

**Survey weighted model is adjusted for clustering and survey design

**Table – S2: Comparison of model estimates and precision using Taylor linearised standard errors and robust standard errors ***

| Variables | Taylor linearised** | | Robust standard errors*** | |
|---|---|---|---|---|
| | *OR* | *95% CI* | *OR* | *95% CI* |
| **Age** | | | | |
| 13-19 | 3.66 | (3.27, 4.10) | 3.66 | (3.27, 4.10) |
| 20-24 | 1.16 | (1.07, 1.26) | 1.16 | (1.08, 1.26) |
| 25-29 | 1.00 | -- | 1.00 | -- |
| 30-34 | 1.19 | (1.08, 1.31) | 1.19 | (1.08, 1.31) |
| 35-39 | 1.92 | (1.72, 2.13) | 1.92 | (1.73, 2.13) |
| 40-49 | 2.80 | (2.49, 3.15) | 2.80 | (2.49, 3.15) |
| **Distal factors** | | | | |
| **Place of residence** | | | | |
| Urban | 1.00 | -- | 1.00 | -- |
| Rural | 0.97 | (0.88, 1.07) | 0.97 | (0.88, 1.07) |
| **Religion** | | | | |
| Hindu | 1.00 | -- | 1.00 | -- |
| Buddhist | 0.58 | (0.12, 2.77) | 0.58 | (0.12, 2.77) |
| Christian | 0.58 | (0.46, 0.74) | 0.58 | (0.46, 0.74) |
| Jain | 1.16 | (0.40, 3.36) | 1.17 | (0.41, 3.36) |
| Muslim | 0.64 | (0.59, 0.70) | 0.64 | (0.59, 0.70) |
| Sikh | 1.08 | (0.61, 1.92) | 1.08 | (0.61, 1.92) |
| Other | 0.72 | (0.54, 0.97) | 0.72 | (0.54, 0.97) |
| **Social Group** | | | | |
| Other | 1.00 | -- | 1.00 | -- |
| Scheduled caste | 1.10 | (1.02, 1.18) | 1.10 | (1.02, 1.18) |
| Scheduled tribe | 1.43 | (1.31, 1.56) | 1.43 | (1.31, 1.56) |
| **Wealth index** | | | | |
| Wealthiest | 1.00 | -- | 1.00 | -- |
| Quintile 4 | 0.99 | (0.89, 1.09) | 0.99 | (0.89, 1.09) |
| Quintile 3 | 0.97 | (0.88, 1.07) | 0.97 | (0.88, 1.07) |
| Quintile 2 | 0.97 | (0.89, 1.07) | 0.97 | (0.89, 1.07) |
| Lowest quintile | 0.81 | (0.74, 0.90) | 0.81 | (0.74, 0.90) |
| **Has health scheme** | | | | |
| Yes | 1.00 | -- | 1.00 | -- |
| No | 2.72 | (2.41, 3.07) | 2.72 | (2.41, 3.07) |
| **Intermediate factors** | | | | |
| **Accessed a health facility** | | | | |
| Yes | 1.00 | -- | 1.00 | -- |
| No | 2.93 | (2.75, 3.12) | 2.93 | (2.75, 3.12) |
| **Proximal factors** | | | | |
| **Pregnancy complication or medical comorbidity** | | | | |
| No | 1.00 | -- | 1.00 | -- |
| Yes | 50.2 | (44.5, 56.6) | 50.2 | (44.5, 56.6) |

*Estimates shown are for the final model with adjustment for all distal, intermediate and proximal factors

**The model using taylor linearised standard errors is estimating by adjustment for survey design using *svy* commands.

***The model using robust standard errors is estimated by adjustment for proportion weights.

**Table – S3: Comparison of model estimates using complete-case analysis and multiple imputation by chained equations***

| Variables | Imputed model** | | Complete-case analysis*** | |
|---|---|---|---|---|
| | *OR* | *95% CI* | *OR* | *95% CI* |
| **Age** | | | | |
| 13-19 | 3.68 | (3.29, 4.13) | 3.66 | (3.27, 4.10) |
| 20-24 | 1.17 | (1.08, 1.27) | 1.16 | (1.07, 1.26) |
| 25-29 | 1.00 | -- | 1.00 | -- |
| 30-34 | 1.18 | (1.07, 1.29) | 1.19 | (1.08, 1.31) |
| 35-39 | 1.86 | (1.67, 2.06) | 1.92 | (1.72, 2.13) |
| 40-49 | 2.70 | (2.40, 3.03) | 2.80 | (2.49, 3.15) |
| **Distal factors** | | | | |
| **Place of residence** | | | | |
| Urban | 1.00 | -- | 1.00 | -- |
| Rural | 0.98 | (0.89, 1.07) | 0.97 | (0.88, 1.07) |
| **Religion** | | | | |
| Hindu | 1.00 | -- | 1.00 | -- |
| Buddhist | 0.55 | (0.11, 2.61) | 0.58 | (0.12, 2.77) |
| Christian | 0.64 | (0.51, 0.80_ | 0.58 | (0.46, 0.74) |
| Jain | 1.17 | (0.41, 3.36) | 1.16 | (0.40, 3.36) |
| Muslim | 0.64 | (0.59, 0.70) | 0.64 | (0.59, 0.70) |
| Sikh | 1.09 | (0.61, 1.93) | 1.08 | (0.61, 1.92) |
| Other | 0.72 | (0.53, 0.97) | 0.72 | (0.54, 0.97) |
| **Social Group** | | | | |
| Other | 1.00 | -- | 1.00 | -- |
| Scheduled caste | 1.09 | (1.01, 1.17) | 1.10 | (1.02, 1.18) |
| Scheduled tribe | 1.45 | (1.33, 1.58) | 1.43 | (1.31, 1.56) |
| **Wealth index** | | | | |
| Wealthiest | 1.00 | -- | 1.00 | -- |
| Quintile 4 | 0.99 | (0.90, 1.09) | 0.99 | (0.89, 1.09) |
| Quintile 3 | 0.97 | (0.88, 1.07) | 0.97 | (0.88, 1.07) |
| Quintile 2 | 0.97 | (0.89, 1.07) | 0.97 | (0.89, 1.07) |
| Lowest quintile | 0.83 | (0.75, 0.91) | 0.81 | (0.74, 0.90) |
| **Has health scheme** | | | | |
| Yes | 1.00 | -- | 1.00 | -- |
| No | 2.68 | (2.37, 3.02) | 2.72 | (2.41, 3.07) |
| **Intermediate factors** | | | | |
| **Accessed a health facility** | | | | |
| Yes | 1.00 | -- | 1.00 | -- |
| No | 2.89 | (2.72, 3.09) | 2.93 | (2.75, 3.12) |
| **Proximal factors** | | | | |
| **Pregnancy complication or medical comorbidity** | | | | |
| No | 1.00 | -- | 1.00 | -- |
| Yes | 45.8 | (41.0, 51.2) | 50.2 | (44.5, 56.6) |

*Estimates shown are for the final model with adjustment for all distal, intermediate and proximal factors
** Multiple imputation was performed on the variable *accessing a health facility*. The imputed model uses proportion weighted data with robust standard errors
***The complete-case analysis model is survey weighted with Taylor linearised standard errors

**Figure S1: Study population derivation**

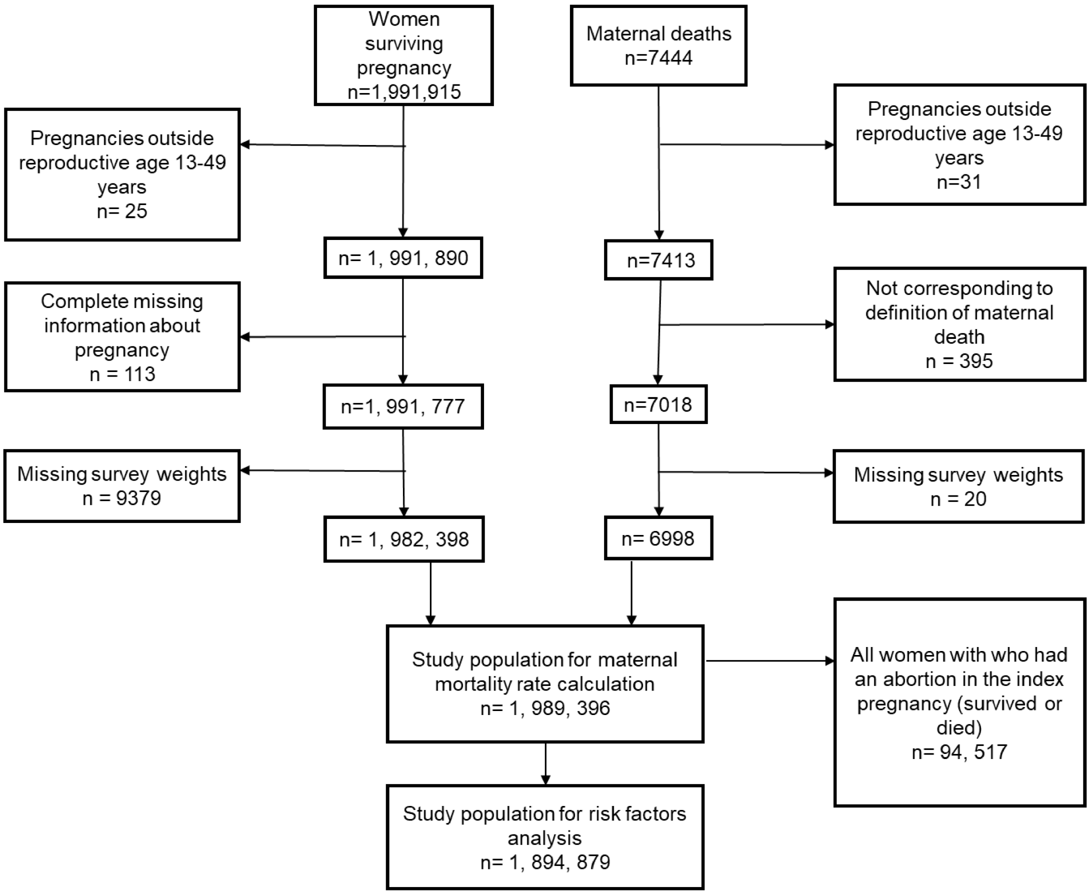

**Figure S2: Theoretical framework for maternal mortality in India**

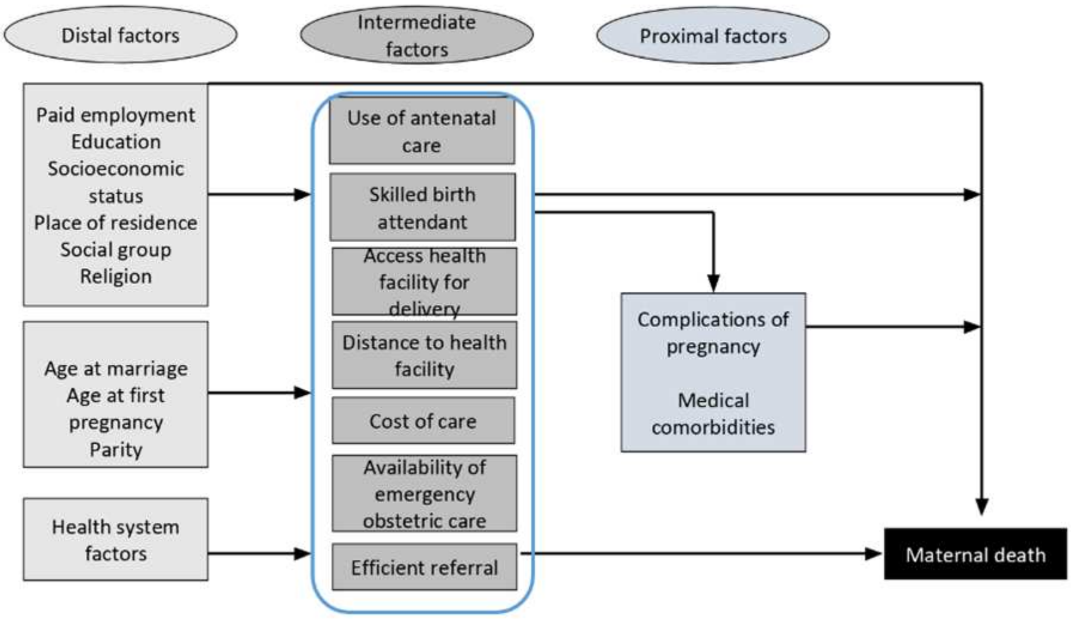

**Figure S3: Odds ratios of maternal mortality according to maternal age with different levels of adjustment**

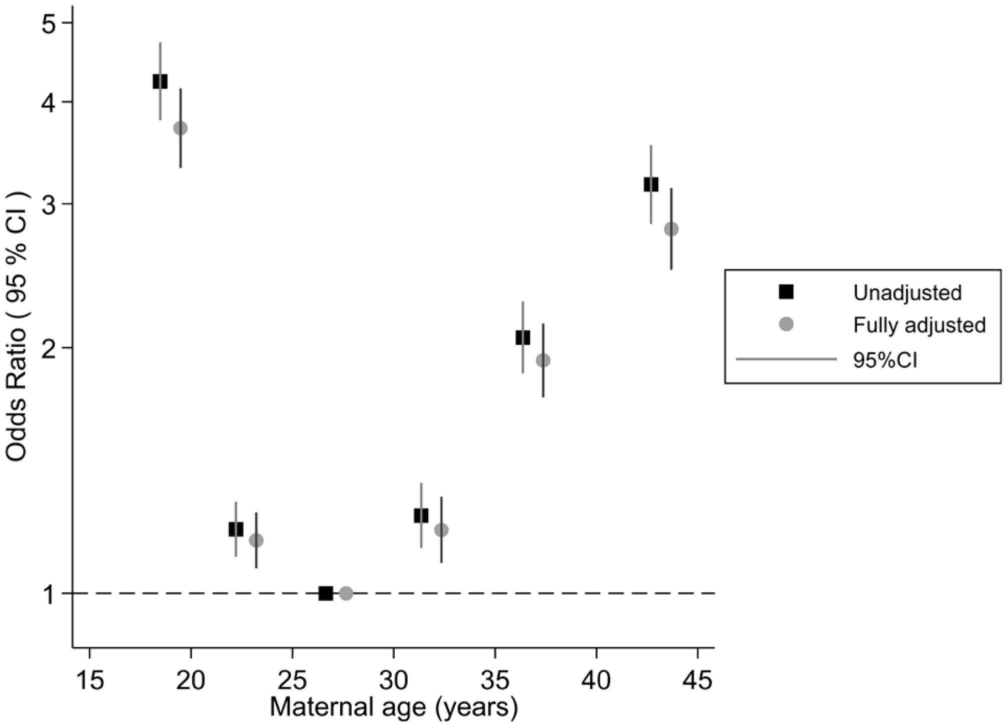


Note: Analyses were performed using logistic regression with adjustment for clustering and survey design. Odds ratios are shown with 95% confidence intervals. The fully adjusted model is fitted for all distal, intermediate and proximal factors.

**Figure S4: Odds ratios of maternal death in rural vs. urban women with sequential adjustment**

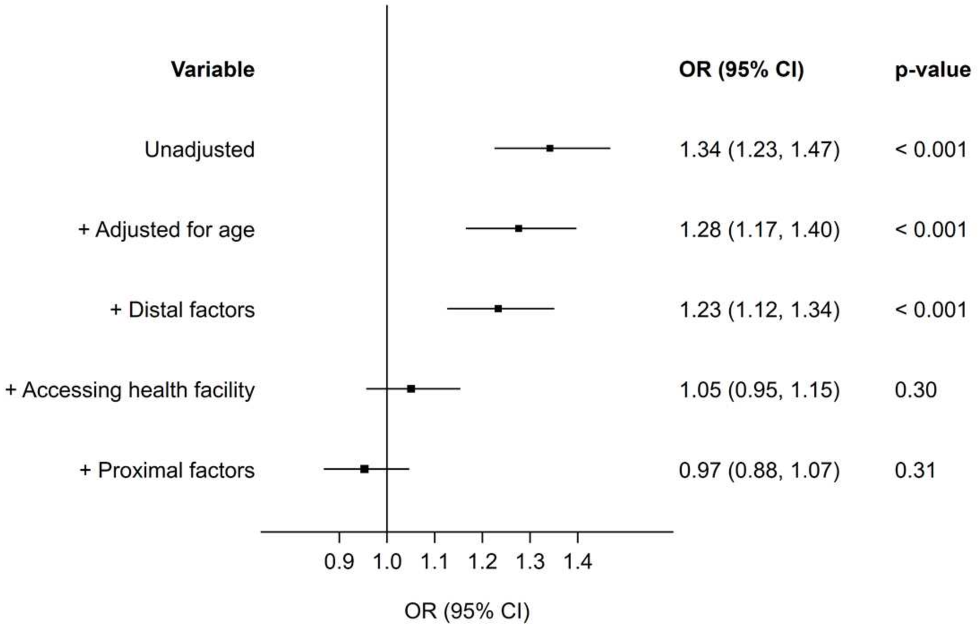


Note : Analyses were performed using logistic regression with adjustment for clustering and survey design. Size of data markers are proportional to the inverse of the variance of the odds ratio.

**Figure S5: Odds ratio of maternal death in poorest vs. wealthiest women with sequential adjustment**

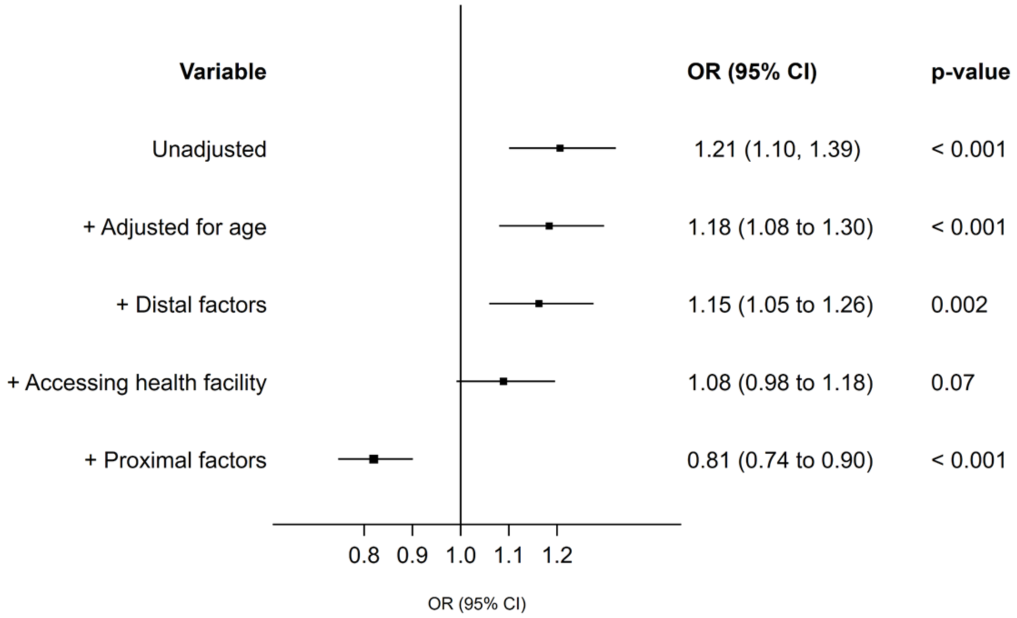


Note : Analyses were performed using logistic regression with adjustment for clustering and survey design. Size of data markers are proportional to the inverse of the variance of the odds ratio.
