# Supplementary material for: Risk factors for maternal mortality among 1.9 million women in nine empowered action group states in India: secondary analysis of Annual Health Survey data
Source: BMJ Open. 2020 Aug 20;10(8):e038910. doi: 10.1136/bmjopen-2020-038910 (PMC7440828; doi:10.1136/bmjopen-2020-038910)
Supplement: Author's manuscript [file bmjopen-2020-038910.draft_revisions.pdf]

BMJ Open

BMJ Open is committed to open peer review. As part of this commitment we make the peer review history of every article we publish publicly available.

When an article is published we post the peer reviewers' comments and the authors' responses online. We also post the versions of the paper that were used during peer review. These are the versions that the peer review comments apply to.

The versions of the paper that follow are the versions that were submitted during the peer review process. They are not the versions of record or the final published versions. They should not be cited or distributed as the published version of this manuscript.

BMJ Open is an open access journal and the full, final, typeset and author-corrected version of record of the manuscript is available on our site with no access controls, subscription charges or pay-per-view fees (http://bmjopen.bmj.com).

If you have any questions on BMJ Open's open peer review process please email info.bmjopen@bmj.com

BMJ Open

Risk factors for maternal mortality among 1.9 million women in nine Empowered Action Group states in India: secondary analysis of Annual Health Survey data

Journal:	*BMJ Open*
Manuscript ID	bmjopen-2020-038910
Article Type:	Original research
Date Submitted by the Author:	31-Mar-2020
Complete List of Authors:	Horwood , Geneviève ; University of Oxford, Nuffield Department of Population Health<br>Opondo, Charles; University of Oxford, Nuffield Department of Population Health<br>Choudhury , Saswati; Gauhati Medical College and Hospital, Department of Obstetrics and Gynaecology<br>Rani, Anjali; Banaras Hindu University Institute of Medical Sciences, Department of Obstetrics and Gynaecology<br>Nair, Manisha; University of Oxford, NPEU, Nuffield Department of Population Health
Keywords:	EPIDEMIOLOGY, OBSTETRICS, PUBLIC HEALTH

SCHOLARONE™
Manuscripts

I, the Submitting Author has the right to grant and does grant on behalf of all authors of the Work (as defined in the below author licence), an exclusive licence and/or a non-exclusive licence for contributions from authors who are: i) UK Crown employees; ii) where BMJ has agreed a CC-BY licence shall apply, and/or iii) in accordance with the terms applicable for US Federal Government officers or employees acting as part of their official duties; on a worldwide, perpetual, irrevocable, royalty-free basis to BMJ Publishing Group Ltd ("BMJ") its licensees and where the relevant Journal is co-owned by BMJ to the co-owners of the Journal, to publish the Work in this journal and any other BMJ products and to exploit all rights, as set out in our licence.

The Submitting Author accepts and understands that any supply made under these terms is made by BMJ to the Submitting Author unless you are acting as an employee on behalf of your employer or a postgraduate student of an affiliated institution which is paying any applicable article publishing charge ("APC") for Open Access articles. Where the Submitting Author wishes to make the Work available on an Open Access basis (and intends to pay the relevant APC), the terms of reuse of such Open Access shall be governed by a Creative Commons licence – details of these licences and which Creative Commons licence will apply to this Work are set out in our licence referred to above.

Other than as permitted in any relevant BMJ Author's Self Archiving Policies, I confirm this Work has not been accepted for publication elsewhere, is not being considered for publication elsewhere and does not duplicate material already published. I confirm all authors consent to publication of this Work and authorise the granting of this licence.

For peer review only

Risk factors for maternal mortality among 1.9 million women in nine Empowered Action Group states in India: secondary analysis of Annual Health Survey data

Authors

1. Geneviève Horwood (MSc)
   MSc student in Global Health Science, Nuffield Department of Population Health, University of Oxford
   Richard Doll Building, Old Road Campus, Headington, Oxford, OX3 7LF, UK
   Email: genevieve.horwood@gmail.com

2. Charles Opondo (PhD)
   National Perinatal Epidemiology Unit, Nuffield Department of Population Health, University of Oxford
   Richard Doll Building, Old Road Campus, Headington, Oxford, OX3 7LF, UK
   Email: charles.opondo@npeu.ox.ac.uk

3. Saswati S Choudhury (MD)
   Department of Obstetrics and Gynaecology, GMCH, Guwahati
   Narkashur Hilltop, Christian Basti Bhangagarh, Guwahati, Assam 781032, India
   Email: saswatischoudhury@gmail.com

4. Anjali Rani (MD)
   Department of Obstetrics and Gynaecology, Institute of Medical Sciences, Banaras Hindu University,
   Ajagara, Banaras Hindu University Campus, Varanasi, Uttar Pradesh 221005, India
   Email: anjalirani.imsbhu@gmail.com

5. Manisha Nair (DPhil)
   National Perinatal Epidemiology Unit, Nuffield Department of Population Health, University of Oxford
   Richard Doll Building, Old Road Campus, Headington, Oxford, OX3 7LF, UK
   Email: manisha.nair@npeu.ox.ac.uk

Corresponding author

Manisha Nair
Senior Epidemiologist and Research Fellow,
National Perinatal Epidemiology Unit, NDPH, University of Oxford
Richard Doll Building, Old Road Campus, Headington, Oxford, OX3 7LF, UK
Email: manisha.nair@npeu.ox.ac.uk; Phone: +44-(0)1865-617820

For peer review only - http://bmjopen.bmj.com/site/about/guidelines.xhtml

ABSTRACT

Objective: To examine the risk factors for pregnancy-related death in India's nine Empowered Action Group States (EAG).

Design: Secondary data analysis of the Indian Annual Health Survey (2010-13)

Setting: Nine states: Assam, Bihar, Chhattisgarh, Jharkhand, Madhya Pradesh, Odisha, Rajasthan, Uttar Pradesh and Uttarakhand.

Participants: 1,989,396 pregnant women.

Methods: Maternal mortality ratio (MMR), overall and for each state, with 95% Confidence Interval (CI) were calculated. Stepwise multivariable logistic regression was used to investigate the association of risk factors with maternal mortality. Area under the receiver-operator characteristic curve (AUC) was used to assess the prediction of the model.

Outcome measures: MMR, adjusted Odds Ratio(aOR), area under curve (AUC), all with 95% CIs.

Results: MMR calculated for the nine states was 383/100,000 live births. Age exhibited a U-shaped association with maternal mortality. Not having a health scheme and belonging to a scheduled caste or scheduled tribe group were significant risk factors for maternal death with aOR of 2.72 (95%CI:2.41-3.07), 1.10 (95%CI:1.02-1.18) and 1.43(95%CI:1.31-1.56), respectively. Socioeconomic status and rural residence were not associated with maternal mortality after adjusting for access to a healthcare facility. Complications of pregnancy and medical comorbidities were the strongest risk factors for maternal death (aOR 50.2, 95% CI:44.5-56.6). Together, risk factors identified accounted for 89%(95%CI:0.887-0.894) of the AUC.

Conclusions: Maternal mortality in India's EAG states greatly exceeds the national average. The identified risk factors demonstrate the importance of improving quality of pregnancy care. Notable, the study showed that the risk conferred by poor socioeconomic status could be mitigated by universal access to healthcare during pregnancy and childbirth.

ARTICLE SUMMARY

Strengths and limitations of this study

- This study identifies the risk factors for maternal mortality in nine socioeconomically disadvantaged states in India, a country with the highest number of maternal deaths, globally.

- The study examines >7000 maternal deaths in a vulnerable population of >1.9 million pregnant women using information about a wide range of risk factors from the Indian Annual Health Survey (2010-2013).

For peer review only - http://bmjopen.bmj.com/site/about/guidelines.xhtml

- The focus of this study was on high burden disadvantaged states, thus the findings may not be generalizable to the rest of India.
- The survey design likely introduced non-systematic reporting bias leading to potential under-reporting of maternal deaths in early pregnancy and deaths due to indirect causes.

Keywords: Maternal death, India, risk factors, pregnancy

Word count: 3240

For peer review only

For peer review only - http://bmjopen.bmj.com/site/about/guidelines.xhtml

INTRODUCTION

Despite advances in modern medicine, pregnancy and childbirth remain one of the leading causes of mortality worldwide for women of reproductive age[1]. The question of safe motherhood is not merely one of public health concern but has been widely recognised as an issue of social injustice[2 3]. According to the Sustainable Development Goals, the global target is to reduce the maternal mortality ratio (MMR) to less than 70 per 100,000 live births by 2030 and to provide universal access to reproductive health care[4].

India is responsible for the second highest number of maternal deaths worldwide(5). India's maternal mortality ratio has been steadily declining since the 1990s[1 5 6] but within-country variations in maternal mortality remain staggering. In the southern state of Kerala, the MMR was reported to be as low as 61 per 100 000 live births in 2013 whereas in the northern state of Bihar it was 208 per 100 000 live births[5]. This inequality in the burden of maternal deaths is reflected by other socio-economic indicators such as poverty and education[7]. Recognising the need to improve maternal heath care in the socioeconomically disadvantaged states, the government of India launched a National Rural Health Mission in 2005, concentrating its efforts on nine Empowered Action Group States (EAG)[8 9]. These are Assam, Bihar, Chhattisgarh, Jharkhand, Madhya Pradesh, Odisha, Rajasthan, Uttar Pradesh and Uttarakhand. Nevertheless, little is known about the population level risk factors that influence maternal death in the vulnerable population in these states. The objective of this study was to examine the risk factors for pregnancy-related death in India's nine EAG states using a large-scale population-based dataset.

METHODS

Study design

This study is a secondary data analysis of the Annual Health Survey (AHS), a government household survey conducted between 2010 and 2013 in the nine EAG states.

Data Source

For peer review only - http://bmjopen.bmj.com/site/about/guidelines.xhtml

The Annual Health Survey (AHS) is a large household survey of over 4 million households[4]. The survey was conducted every year between 2010 and 2013 in the nine EAG states. Compared with the Demographic and Household surveys, AHS collected more detailed information on events and outcomes related to women's reproductive health and pregnancy occurring between the 1st January 2007 and the 31st of December 2009[9]. The survey used a stratified simple random sampling strategy. It recorded information regarding demographic and socioeconomic characteristics, reproductive and sexual health information, and maternal death.

For peer review only

Study Sample

The study included 1,991,915 women reporting a pregnancy within the defined survey time frame and 7444 pregnancy-related deaths as reported by a member of the same household. Women were excluded if their age was outside the reproductive window. While the WHO considers the reproductive age to be 15-49 years, pregnancies occurring at age 13 and above were considered plausible and were included in the analysis. Women with missing survey weights or missing pregnancy outcomes were excluded. In the mortality data set, deaths occurring outside the time frame included in the definition of maternal death (see box 1) were excluded. The survey did not ask household members to identify factors associated with death in the case of women dying after an abortion. As such, all women reporting an abortion were removed from the risk factor analysis. Figure S1 (Supplementary file) depicts the study sample derivation.

Box 1: Definitions related to maternal death

**Maternal death**: Death of a woman while pregnant or within 42 days of termination of pregnancy, irrespective of the duration and site of the pregnancy, from any cause related to or aggravated by the pregnancy or its management, but not from accidental or incidental causes[5].
**Maternal mortality ratio**: Number of maternal deaths per 100 000 livebirths[5].

For peer review only - http://bmjopen.bmj.com/site/about/guidelines.xhtml

Variable choice and extraction

A systematic search of the literature was undertaken by interrogating the Medline, Embase and Global Health databases as well as the WHO's list of publications, to identify potential risk factors for maternal death in India. Using the literature search, hypothesized predictors of maternal mortality specific to our study setting were divided into broad categories of distal, intermediate and proximal factors. The theoretical framework is depicted in Figure S2.

This theoretical framework guided the choice of variables used in the statistical analysis. The mortality questionnaire was brief and answered by proxies of deceased women, so few of the variables identified by the literature review were available. Of the distal factors identified in the framework, those available were place of residence, wealth index, having a health scheme, religion and social group *(categorised as 'Schedule caste (SC)', 'Schedule tribe (ST)' and 'Others'; SC and ST are considered socioeconomically disadvantaged groups)*. Of the intermediate factors, the only variable available was accessing a health facility. For the proximal factors, a binary variable was created distinguishing between women with any complication of pregnancy or medical comorbidity and women with neither. Comorbidities included hypertensive disorders of pregnancy, sepsis, bleeding, jaundice, obstructed or prolonged labour, other pregnancy complications, and other medical conditions.

Statistical analysis

Frequency distributions of binary and categorical variables as well as measures of central tendency for continuous variables were reported. Baseline characteristics were assessed and contrasted between maternal deaths and all surviving women. Frequency distributions of maternal deaths by identified delays to care were examined. The maternal mortality ratio (defined in Box-1) was calculated in each state and for the region overall using survey weighted counts of maternal deaths and live births.

For peer review only - http://bmjopen.bmj.com/site/about/guidelines.xhtml

The univariable association of each potential risk factor with maternal death was explored using simple logistic regression. Stepwise logistic regression was then used to build a multivariable model. The sequential addition of variables to the model was informed by the theoretical framework, where distal factors were added first, followed by intermediate and proximal factors. Statistical interaction between social group and wealth quintiles was tested. To inform appropriate modelling of the effect of age, tests for departure from linearity and trend were conducted. If the effects of risk factors were found to be markedly attenuated after full adjustment, they were explored further by assessing confounding of the association by distal, intermediate, and proximal factors.

Observations were not expected to be independent within clusters, therefore analyses were adjusted for clustering and sampling design. Wald test estimation-values were reported and a modified Hosmer-Lemeshow test, appropriate for clustered data, was used to assess model goodness of fit[10]. To assess the potential effect of clustering on the variance of the model estimates, standard errors were compared between models using Taylor linearised estimates of the variance and models using robust standard errors. In addition, point estimates and confidence intervals were compared between models with adjustment for clustering and those without adjustment.

Patterns and amounts of missing data were described for each of the included variables in the final model. Variables included in our analysis had very small proportion of missing data. Therefore, a complete-case analysis was retained in our final model, although a sensitivity analysis was undertaken with multiple imputation by chained equations.

To assess the risk prediction of each of the groups of risk factors (distal, intermediate, and proximal), the areas under the receiver-operator characteristic (AUROC) curves for the models corresponding to the addition of each of these groups were compared.

For peer review only - http://bmjopen.bmj.com/site/about/guidelines.xhtml

All analyses were carried out using Stata 14.2. P-values of less than 0.05 were considered statistically significant.

Patient and Public Involvement

This is not applicable since this was a secondary analysis of anonymous survey data.

RESULTS

Table 1 shows the baseline characteristics of women included in the study. The median age was 26 years and was the same for women surviving pregnancy and women dying in pregnancy. In general, the households with maternal deaths were more likely to be from a rural area, to be from the two lower wealth quintiles, to belong to a scheduled caste or scheduled tribe social group and to not have a health scheme. Of the women surviving pregnancy, 53.1% accessed a health facility compared to only 36.1% of women dying in pregnancy. In addition, 92.4% of the women dying in pregnancy suffered at least one complication of pregnancy, compared to 26.3% of women surviving pregnancy.

Maternal mortality ratio

The overall maternal mortality ratio was 383 per 100, 000 live births. Most states had an MMR close to but just below the regional estimate. Two states had an MMR of over 500. Assam had an MMR of 552 per 100, 000 live births and Odisha 534 per 100,000 live births. The state with the lowest MMR was Uttarakhand accounting for 209 maternal deaths per 100, 000 live births. No state had an MMR below 200 (Figure 1).

Delays contributing to maternal death

Figure 2 depicts maternal death according to the identified delay most contributing to the death. The delay most frequently cited was inappropriate quality of care at the health facility (55.1%). Lack of care seeking was the second most important delay (18.1%), followed by difficulty in reaching the facility (15.3%). In the groups expressing a delay in care seeking or in reaching the

For peer review only - http://bmjopen.bmj.com/site/about/guidelines.xhtml

facility, over 80% had not registered the death. Conversely, in those expressing a delay in quality of care, 57% had registered the death.

For peer review only

For peer review only - http://bmjopen.bmj.com/site/about/guidelines.xhtml

Table 1: Baseline characteristics of the women included in the study population*		
	**Surviving women**	**Maternal deaths**
**N**	1982398	6998
**Age (yrs)****	26.0 (23.0, 30.0)	26.0 (22.0, 32.0)
**State**		
Assam	168 040 (5%)	812 (7.1%)
Bihar	353 043 (18.5%)	1247 (18.6%)
Chhattisgarh	106 416 (4.1%)	335 (3.5%)
Jharkhand	162 987 (5.3%)	647 (4.8%)
Madhya Pradesh	233 964 (12.0%)	742 (11.2%)
Odisha	121 777 (4.7%)	649 (6.5%)
Rajasthan	150 668 (9.1%)	529 (8.9%)
Uttar Pradesh	538 160 (39.9%)	1778 (38.6%)
Uttarakhand	147 343 (1.5%)	259 (0.8%)
**Region of residence**		
Rural	1 690 156 (82.8%)	6118 (85.5%)
Urban	292 242 (17.2%)	738 (13.1%)
Missing§	0	142 (1.5%)
**Religion**		
Buddhist	956 (<0.01%)	2 (<0.01%)
Christian	26 109 (1.1%)	114 (1.2%)
Hindu	1 632 888 (81.4%)	5813 (83.0%)
Jain	2074 (0.1%)	4 (<0.01%)
Muslim	292 640 (16.4%)	903 (13.9%)
Sikh	4713 (0.2%)	14 (0.2%)
Other	21 206 (0.5%)	83 (0.6%)
Missing§	1812 (0.1%)	65 (1.1 %)
**Social group**		
Scheduled caste	386 169 (20.6%)	1533 (23.3%)
Scheduled tribe	244 057 (10.5%)	1082 (12.2%)
Others	1 350 426 (68.7%)	4318 (63.4%)
Missing§	1746 (0.1%)	65 (1.1%)
**Has health scheme**		
No	1 826 156 (92.7%)	6552 (93.9%)
Yes	15 6242 (7.3%)	446 (6.1%)
**Wealth index quintile**		
Poorest	397 828 (20.1%)	1492 (21.9%)
2	397 338 (21.8%)	1588 (23.9%)
3	397 600 (20.4%)	1400 (20.6%)
4	395 890 (18.7%)	1269 (17.5%)
Wealthiest	393 742 (18.3%)	1249 (16.1%)
**Accessing a health facility**		
Yes	1 066 136 (53.1%)	2579 (36.1%)
No	798 811 (40.1%)	4322 (63.0%)
Missing§	117 451 (6.01%)	97 (0.9%)
**Pregnancy complication or comorbidity**		
No	1 460 887 (73.0%)	535 (6.6%)
Yes	521 511 (27.0%)	6463 (93.4%)

* Characteristics are presented as unweighted counts and survey weighted frequencies. ** Age is presented as the median with interquartile range. §Missing reported here includes women with abortion-related deaths who were not included in the final model for risk factor analysis.

For peer review only - http://bmjopen.bmj.com/site/about/guidelines.xhtml

Risk factors for maternal mortality

There was a departure from linearity in the association between age and maternal death and as such, age was coded as a categorical variable. Women between the ages of 25 to 29 years were least likely to die during pregnancy. The youngest age group, women between 13 and 19 years, was the group with the highest risk of death in pregnancy (adjusted odds ratio (aOR) 3.66, 95% CI 3.27-4.10), followed by the eldest group of women of between 40 and 49 years of age (aOR 2.80, 95% CI 2.49-3.15). Age exhibited a strong u-shaped relationship with maternal death and this relationship was largely unchanged after full adjustment for other risk factors (Figure S3).

After adjustment for all distal factors, the effect size of the association between rural residence, social group, health scheme, and maternal death were all mildly attenuated but remained significant. There was a trend of association across wealth quintile groups and maternal death. Associations between not having a health scheme, accessing a health facility or the presence of a complication with maternal death were all strengthened after adjustment for distal factors (Table 2). However, attenuation of the associations of place of residence, wealth index and social group with maternal death occurred after adjustment for the presence of a complication or medical comorbidity (Table 2). Being of a scheduled caste or scheduled tribe group continued to be significantly associated with maternal death, exhibiting an aOR 1.43, 95% CI 95%CI 1.31-1.56. Not having a health scheme conferred significant risk (aOR 2.72, 95%CI 2.41-3.07) after adjustment for other factors.

After taking into account the access to a health facility, the association between rural residence and maternal death was attenuated (aOR 1.05, 95% CI 0.95-1.15). In addition, the trend of association between wealth quintile groups and maternal death was no longer significant and there was no evidence of association between wealth index and dying in pregnancy. Figures S4 and S5 depict the attenuation of the association between maternal mortality and both wealth and rural residence with sequential adjustment for other risk factors.

For peer review only - http://bmjopen.bmj.com/site/about/guidelines.xhtml

Table 2: Risk factors for maternal mortality with progressive adjustment

Variables	Unadjusted		Adjusted for age		Distal factors*		Intermediate factors†		Proximal factors‡	
	OR	*95% CI*	*OR*	*95% CI*	*OR*	*95% CI*	*OR*	*95% CI*	*OR*	*95% CI*
**Age (years)**										
13-19	4.23	(3.80, 4.73)	NA	--	4.12	(3.69, 4.62)	4.34	(3.87, 4.85)	3.66	(3.27, 4.10)
20-24	1.20	(1.11, 1.30)	NA	--	1.20	(1.11, 1.30)	1.25	(1.15, 1.35)	1.16	(1.07, 1.26)
25-29	1.00	--	NA	--	1.00	--	1.00	--	1.00	--
30-34	1.25	(1.14, 1.37)	NA	--	1.25	(1.14, 1.37)	1.20	(1.10, 1.32)	1.19	(1.08, 1.31)
35-39	2.06	(1.86, 2.27)	NA	--	2.03	(1.83, 2.25)	1.91	(1.72, 2.12)	1.92	(1.72, 2.13)
40-49	3.17	(2.83, 3.54)	NA	--	3.13	(2.79, 3.51)	2.91	(2.60, 3.27)	2.80	(2.49, 3.15)
**Distal factors**										
**Place of residence**										
Urban	1.00	--	1.00	--	1.00	--	1.00	--	1.00	--
Rural	1.34	(1.23, 1.47)	1.28	(1.17, 1.40)	1.23	(1.12, 1.34)	1.05	(0.95, 1.15)	0.97	(0.88, 1.07)
**Religion**										
Hindu	1.00	--	1.00	--	1.00	--	1.00	--	1.00	--
Buddhist	0.43	(0.09, 2.01)	0.45	(0.10, 2.12)	0.46	(0.10, 2.14)	0.52	(0.11, 2.46)	0.58	(0.12, 2.77)
Christian	1.03	(0.82, 1.30)	1.01	(0.80, 1.26)	1.02	(0.81, 1.28)	0.90	(0.70, 1.14)	0.58	(0.46, 0.74)
Jain	0.58	(0.20, 1.65)	0.63	(0.22, 1.79)	0.77	(0.27, 2.20)	0.91	(0.32, 2.61)	1.16	(0.40, 3.36)
Muslim	0.83	(0.76, 0.91)	0.79	(0.73, 0.86)	0.84	(0.77, 0.92)	0.76	(0.69, 0.83)	0.64	(0.59, 0.70)
Sikh	0.86	(0.48, 1.52)	0.94	(0.53, 1.67)	1.03	(0.58, 1.82)	1.12	(0.63, 1.99)	1.08	(0.61, 1.92)
Other	1.03	(0.78, 1.37)	0.96	(0.72, 1.27)	0.88	(0.66, 1.18)	0.76	(0.56, 1.02)	0.72	(0.54, 0.97)
**Social Group**										
Other	1.00	--	1.00	--	1.00	--	1.00	--	1.00	--
Scheduled caste	1.22	(1.14, 1.31)	1.20	(1.12, 1.29)	1.15	(1.06, 1.23)	1.10	(1.02, 1.19)	1.10	(1.02, 1.18)
Scheduled tribe	1.24	(1.14, 1.34)	1.19	(1.10, 1.29)	1.15	(1.05, 1.26)	1.11	(1.02, 1.21)	1.43	(1.31, 1.56)
**Wealth index**										
Wealthiest	1.00	--	1.00	--	1.00	--	1.00	--	1.00	--
Quintile 4	1.07	(0.97, 1.17)	1.07	(0.97, 1.17)	1.07	(0.97, 1.18)	1.03	(0.94, 1.14)	0.99	(0.89, 1.09)
Quintile 3	1.16	(1.06, 1.27)	1.14	(1.04, 1.25)	1.17	(1.06, 1.28)	1.09	(0.99, 1.20)	0.97	(0.88, 1.07)
Quintile 2	1.25	(1.14, 1.37)	1.21	(1.11, 1.32)	1.22	(1.11, 1.33)	1.11	(1.02, 1.22)	0.97	(0.89, 1.07)
Lowest quintile	1.21	(1.10, 1.32)	1.18	(1.08, 1.30)	1.15	(1.05, 1.26)	1.08	(0.98, 1.18)	0.81	(0.74, 0.90)
**Has health scheme**										
Yes	1.00	--	1.00	--	1.00	--	1.00	--	1.00	--
No	1.24	(1.10, 1.39)	1.26	(1.12, 1.41)	1.36	(1.21, 1.53)	1.33	(1.18, 1.50)	2.72	(2.41, 3.07)
**Intermediate factors**										
**Accessed health facility**										
Yes	1.00	--	1.00	--	1.00	--	NA	--	1.00	--
No	2.26	(2.13, 2.40)	2.21	(2.09, 2.35)	2.22	(2.08, 2.36)	NA	--	--	2.93
**Proximal factors****										
No	1.00	--	1.00	--	1.00	--	1.00	--	1.00	--
Yes	36.5	(32.8, 40.7)	36.2	(32.6, 40.3)	40.1	(35.9, 44.8)	50.2	(44.5, 56.6)	NA	--

For peer review only - http://bmjopen.bmj.com/site/about/guidelines.xhtml

§ All analyses use logistic regression and are adjusted for clustering and survey design; * Further adjusted for distal factors †Further adjusted for accessing health facility ‡Further adjusted for proximal factors; ** Pregnancy complication or medical comorbidity

For peer review only

For peer review only - http://bmjopen.bmj.com/site/about/guidelines.xhtml

The magnitude of the association between proximal factors (pregnancy complications and medical comorbidities) and maternal death was further increased after adjustment for the distal and intermediate factors (aOR 50.2, 95% CI 44.5-56.6).

F-statistics adjusted for survey design as a measure of contribution to the model fit showed that the variables accounting for the greatest amount of variation in the data were proximal factors, accessing a health facility, not having a health scheme, and age. Model diagnostics showed no evidence of collinearity between dependent variables or model misspecification.

In assessing clustering of the data, standard errors were compared between models with survey weighted data using Taylor linearization variance estimates and proportion weighted data using robust standard errors. These were found to be equivalent. Point estimates and standard errors were similar between models with unweighted data and survey weighted data. These findings suggested that clustering effects were minimal in the study population. Further, the findings were comparable in imputed models and complete-case analysis. See Tables S1, S2 and S3 in the supplementary file.

Figure 3 shows the area under the curve of the receiver-operator characteristic curve for each group of risk factors. Together, the distal and intermediate factors accounted for 66% (95% CI 0.649-0.662) of the variance in the data. With the addition of the proximal factors, this increased to 89% (95% CI 0.887-0.894).

For peer review only

DISCUSSION

Our study showed that the maternal mortality ratio in the nine states was much higher than the corresponding WHO estimates for India's overall MMR for a similar time-period. Lack of quality care in the health facilities were perceived as the factor most contributing to the maternal deaths by family members of deceased women. Proximal factors, which encompassed any pregnancy

For peer review only - http://bmjopen.bmj.com/site/about/guidelines.xhtml

complication or medical comorbidity, were the strongest predictors of women dying in pregnancy followed by lack of access to health facility. More importantly, the study showed that the effects of living rurally and being poor could be mitigated by improving access to health facility. Other risk factors identified were younger or older maternal age, belonging to a scheduled tribe or caste social group, and not being enrolled in a health scheme.

The WHO reported MMR for India in 2010 was 208/100,000 live births[1 6] compared with 383/100,000 live births calculated in our study population for a similar period (2007-2009). This discrepancy reflects the high burden of maternal mortality in India's nine socioeconomically disadvantaged states. Moreover, in our study, more than half of the maternal deaths had not been previously recorded. Efforts to create a national electronic database for maternal death surveillance in India began in 2013[11], but the progress is still limited, mainly due to lack of resources[12 13].

Our study identified two key modifiable health systems determinants of maternal mortality in the nine disadvantaged states – access to health facility and quality of care. Research in many low-and-middle-income countries (LMICs) consistently show that living in rural areas and being poor are associated with decreased odds of childbirth in health facility, antenatal care, and skilled attendance at birth[14 15]. Our study demonstrated that the effects of wealth and living rurally were mediated by a woman's ability to access care and do not exert a direct effect on maternal mortality. This is an important finding in the context of India and other LMICs in terms of universal access to antenatal, delivery and postnatal care. Access to appropriate good quality care could mitigate the effects of socioeconomic inequalities know to be associated with a high burden of maternal mortality.

The ROC curve analysis demonstrates convincingly that proximal factors (pregnancy complications and medical co-morbidities) were the strongest predictors of maternal mortality in this population. Further, the association between proximal factors and maternal death was

For peer review only - http://bmjopen.bmj.com/site/about/guidelines.xhtml

strengthened after accounting for other factors showing that these complications remain the most important risk factors for maternal death, thus reinforcing the importance of high quality antenatal care, availability of basic emergency obstetrical care, and postnatal care. Nonetheless, in our study population, poor quality of care was reported as the factor most contributing to the maternal death in 55% of the deaths.

In 2005, the Indian government implemented the National Heath Mission to address gaps in maternal health care. Improving the access to and quality of care, particularly for rural areas in high focus states, were the main objectives of this initiative[8]. Since 2005, India has seen some improvements. Institutional deliveries increased from 39% in 2005 to 79% in 2013. Complete antenatal care coverage increased from 37 to 51% and postnatal care increased from 27 to 36%[16]. However, studies show that the programme has had little or no effect on clinical outcomes. For example, despite the increase in health facility deliveries, there has been no demonstrated decline in maternal mortality[17 18].

Studies have repeatedly shown that quality of care is lacking in public health facilities including non-availability of essential medicines such as uterotonics and antibiotics, and lack of facilities for caesarean section and blood transfusion[19]. Further, government reports on the state of public facilities in these nine states have reported lack of toilet facilities, unreliable electricity, and unsatisfactory cleanliness[20], and lack of basic measures of obstetrical care such as adequate hand hygiene[21]. Moreover, there have been reports of verbal and physical abuse of staff towards patients[20 22].

Conforming with the findings of other studies[23 24], age and maternal death exhibited a strong u-shaped association in which the youngest (women aged 13 to 19 years) and the oldest age group (women aged 40-49 years) had the highest risk of dying. India's National Family Health Survey showed that of women aged 20 to 49, 27% had been married before the age of 15 and 58% were married before the legal age of marriage of 18 years. In addition, 30% of Indian

For peer review only - http://bmjopen.bmj.com/site/about/guidelines.xhtml

women had given birth by age 19[16]. While age itself is not a modifiable risk factor, childbearing-age should be regarded as one. Women should be able to exert control over when they choose to become pregnant. Policies supporting universal access to contraception, family planning counselling, safe abortion, and promoting women's empowerment need continued attention in India.

This is a large population-based study conducted using a survey with minimal sampling bias and included a sample size of almost two million women and over 7000 recorded maternal deaths. The AHS database had two advantages over other household surveys in India: (i) it had more detailed information about a range of potential risk factors that we set out to examine in this study, and (ii) it allowed us to focus our analysis on socioeconomically disadvantaged states with a high burden of maternal deaths. We were able to study a population where maternal deaths are often unreported and where vital statistics and health related data are lacking. One limitation is that the AHS was conducted in 2010-13 and it is possible that the risk factors identified in our study may have changed over the years. However, they are unlikely to have changed significantly considering that these nine states continue to have a higher maternal mortality rate than the rest of India. The risk factors included in the regression model were informed by both established theory in the field of maternal health and a current review of the literature specific to the study setting, and the analysis addressed distal, intermediate, and proximal causes of maternal death crucial to the understanding of women's health dynamics in the most vulnerable population in India's Empowered Action Group states.

We acknowledge that information collected about women dying in pregnancy was limited and did not include several factors identified as important by the literature review such as education, paid employment, parity, age at first pregnancy, distance to the health facility or having a skilled birth attendant. However, a majority of these factors would have been covered by the 'wealth index' and 'access to health facility'. The survey design is also likely to have introduced non-systematic reporting bias. In keeping with previous literature, underreporting of early pregnancy deaths,

For peer review only - http://bmjopen.bmj.com/site/about/guidelines.xhtml

particularly those arising from complications of abortion, and of maternal deaths arising from indirect causes could have led to an underestimation of maternal deaths[25].

Maternal mortality in India's Empowered Action Group states greatly exceeds the national average and is far from the target set out by the Sustainable Development Goals (SDGs). Analysis of the results in the context of India's health care system suggests that targeted interventions to improve maternal outcomes in this population would include improved in-hospital care, improved access to healthcare services, and guaranteed skilled personnel and basic emergency obstetrical care in every maternity centre. Reducing maternal mortality in India's socioeconomically disadvantaged states is achievable but will require continued accelerated national efforts to recognise the importance of women's lives and invest in sustainable health systems founded on principles of accountability and evidence-based medicine.

REFERENCES

1. Alkema L, Chou D, Hogan D, et al. Global, regional, and national levels and trends in maternal mortality between 1990 and 2015, with scenario-based projections to 2030: a systematic analysis by the UN Maternal Mortality Estimation Inter-Agency Group. *Lancet (London, England)* 2016;387(10017):462-74. doi: 10.1016/s0140-6736(15)00838-7 [published Online First: 2015/11/21]
2. Rosenfield A, Maine D. Maternal mortality--a neglected tragedy. Where is the M in MCH? *Lancet (London, England)* 1985;2(8446):83-5. doi: 10.1016/s0140-6736(85)90188-6 [published Online First: 1985/07/13]
3. WHO. Reduction of Maternal Mortality: A Joint WHO/UNFPA/UNICEF World Bank Statement. Geneva: WHO, 1999.
4. World Health Organisation. MDG 5:Improve maternal health Geneva: WHO; 2015 [cited 2020 07 February]. Available from: https://www.who.int/topics/millennium_development_goals/maternal_health/en/ accessed 07 February 2020.
5. Registrar General of India Sample registration system. Special Bulletin on Maternal Mortality in India 2014-16 Sample Registration System. *SRS Bulletin* 2018(91):16–8.
6. WHO. Trends in maternal mortality: 1990 to 2015. Geneva: WHO, 2018.
7. The World Bank Group. Kerala: Indicators at a glance: The World Bank Group, 2017.
8. Chatterjee P. The health system in India: the underserved majority. *Lancet (London, England)* 2017;390(10111):2426-27. doi: 10.1016/s0140-6736(17)32860-x [published Online First: 2017/11/19]
9. Office of the Registrar General & Census Commissioner. Annual Health Survey Report: A report on Core and Vital Health Indicators- Part 1, 2016.

For peer review only - http://bmjopen.bmj.com/site/about/guidelines.xhtml

10. Archer KJ, Lemeshow S, Hosmer DW. Goodness-of-fit tests for logistic regression models when data are collected using a complex sampling design. *Computational Statistics & Data Analysis* 2007;51(9):4450-64. doi: https://doi.org/10.1016/j.csda.2006.07.006
11. Purandare C, Bhardwaj A, Malhotra M, et al. Every death counts: Electronic tracking systems for maternal death review in India. *International Journal of Gynecology & Obstetrics* 2014;127:S35-S39. doi: https://doi.org/10.1016/j.ijgo.2014.09.003
12. Gupta M, Rao C, Lakshmi PVM, et al. Estimating mortality using data from civil registration: a cross-sectional study in India. *Bull World Health Organ* 2016;94(1):10-21. doi: 10.2471/BLT.15.153585 [published Online First: 11/02]
13. Rane TM, Mahanta TG, Islam S, et al. Civil registration system (CRS) for birth and death registration in Assam – A rapid assessment. *Clinical Epidemiology and Global Health* 2019 doi: https://doi.org/10.1016/j.cegh.2019.05.006
14. Ahmed S, Creanga AA, Gillespie DG, et al. Economic Status, Education and Empowerment: Implications for Maternal Health Service Utilization in Developing Countries. *PLOS ONE* 2010;5(6):e11190. doi: 10.1371/journal.pone.0011190
15. Bauserman M, Lokangaka A, Thorsten V, et al. Risk factors for maternal death and trends in maternal mortality in low- and middle-income countries: a prospective longitudinal cohort analysis. *Reproductive health* 2015;12 Suppl 2:S5. doi: 10.1186/1742-4755-12-s2-s5 [published Online First: 2015/06/13]
16. International Institute for Population Sciences. National Family Health Survey (NFHS-4) 2015-16 India *International Institute for Population Sciences (IIPS) and ICF* 2017:1–192. doi: http://rchiips.org/NFHS/NFHS-4Reports/India.pdf
17. Lim SS, Dandona L, Hoisington JA, et al. India's Janani Suraksha Yojana, a conditional cash transfer programme to increase births in health facilities: an impact evaluation. *The Lancet* 2010;375(9730):2009-23. doi: 10.1016/S0140-6736(10)60744-1
18. Randive B, Diwan V, De Costa A. India's Conditional Cash Transfer Programme (the JSY) to Promote Institutional Birth: Is There an Association between Institutional Birth Proportion and Maternal Mortality? *PLoS One* 2013;8(6):e67452. doi: 10.1371/journal.pone.0067452 [published Online First: 2013/07/05]
19. Chaturvedi S, Randive B, Diwan V, et al. Quality of obstetric referral services in India's JSY cash transfer programme for institutional births: a study from Madhya Pradesh province. *PLoS One* 2014;9(5):e96773. doi: 10.1371/journal.pone.0096773 [published Online First: 2014/05/09]
20. Ministry of Health & Family Welfare Government of India. Second Common Review Mission Report, 2008:1–124.
21. Spector JM, Lashoher A, Agrawal P, et al. Designing the WHO Safe Childbirth Checklist program to improve quality of care at childbirth. *International journal of gynaecology and obstetrics: the official organ of the International Federation of Gynaecology and Obstetrics* 2013;122(2):164-8. doi: 10.1016/j.ijgo.2013.03.022 [published Online First: 2013/06/08]
22. Nair H, Panda R. Quality of maternal healthcare in India: Has the National Rural Health Mission made a difference? *J Glob Health* 2011;1(1):79-86.
23. Blanc AK, Winfrey W, Ross J. New Findings for Maternal Mortality Age Patterns: Aggregated Results for 38 Countries. *PLOS ONE* 2013;8(4):e59864. doi: 10.1371/journal.pone.0059864
24. Nove A, Matthews Z, Neal S, et al. Maternal mortality in adolescents compared with women of other ages: evidence from 144 countries. *The Lancet Global Health* 2014;2(3):e155-e64. doi: 10.1016/S2214-109X(13)70179-7
25. Yokoe R, Rowe R, Choudhury SS, et al. Unsafe abortion and abortion-related death among 1.8 million women in India. *BMJ Global Health* 2019;4(3):e001491. doi: 10.1136/bmjgh-2019-001491

For peer review only - http://bmjopen.bmj.com/site/about/guidelines.xhtml

Conflicts of interest: The authors declare that we have no competing interests.

Author statement: GH reviewed the literature, conducted the analysis and wrote the first draft of the paper; CO supervised the data analysis, interpretation and discussion of the results, and edited the paper; SSC helped in acquiring and interpreting the data, and edited the paper; AR edited the paper; MN developed the concept for the study, supervised the data analysis, interpretation and discussion of the results, and edited the paper.

Ethical approval: Ethics approval was not required since this was a secondary analysis of anonymous survey data.

Funding: The study was funded by a Medical Research Council Career Development Award to Manisha Nair (Grant Ref: MR/P022030/1). The funder had no role in the study design, data analysis, data interpretation, or writing of the manuscript. All authors, had full access to all of the data in the study and can take responsibility for the integrity of the data and the accuracy of the data analysis.

Data statement

The anonymised data is freely available through the Indian Government's Data Sharing Portal.

Figure legends

Figure 1: Maternal mortality ratio by state and overall

Figure 2: Frequencies of maternal death according to the delay cited by household members as most contributing to the death

Figure 3: Area under the receiver-operator characteristic curve for distal, intermediate and proximal factors

For peer review only - http://bmjopen.bmj.com/site/about/guidelines.xhtml

Figure 1: Maternal mortality ratio by state and overall

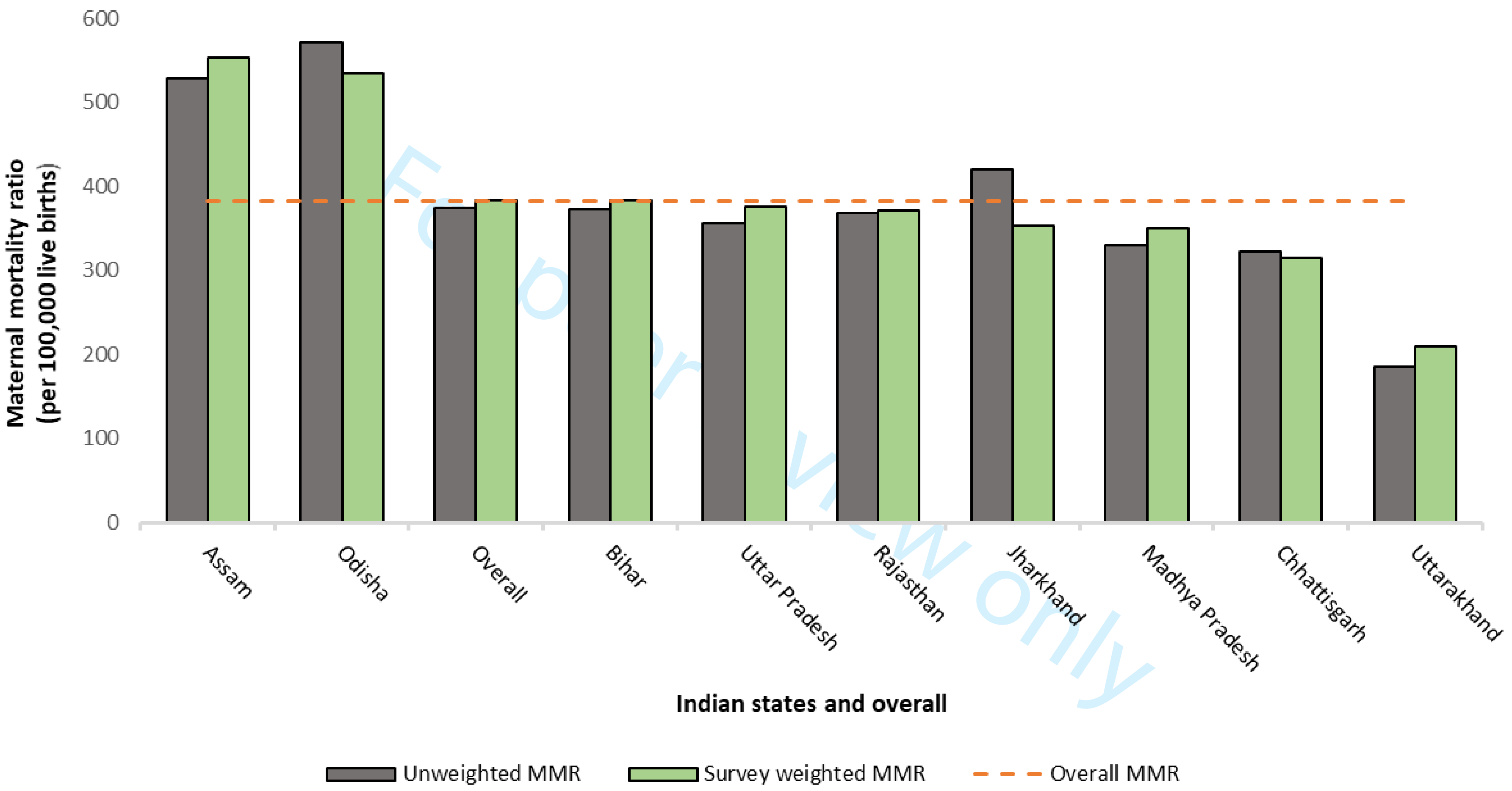

Note: Maternal mortality ratios were calculated using unweighted and survey weighted counts of maternal deaths and live births in each state and for the region overall. States are shown in descending order of survey weighted maternal mortality ratios.

Figure 2: **Frequencies of maternal death according to the delay cited by household members as most contributing to the death**

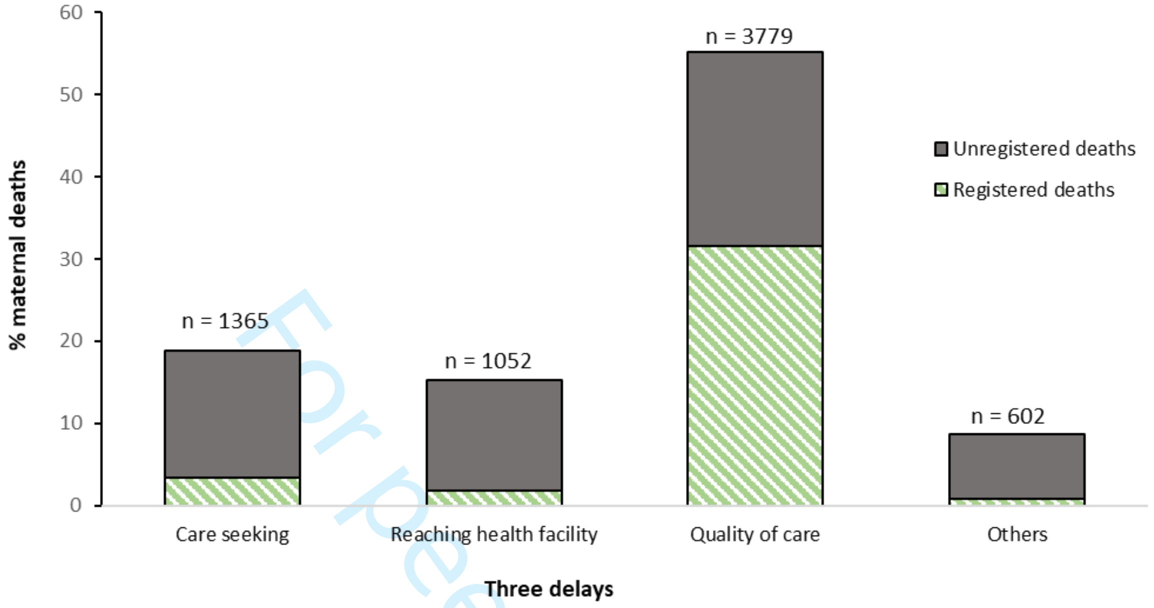

Note: Maternal death frequencies are presented as survey weighted frequencies. Unweighted counts of maternal deaths for each delay are presented over each bar. For each delay, maternal deaths are divided by proportion of registered and unregistered deaths.

For peer review only

Figure 3: Area under the receiver-operator characteristic curve for distal, intermediate and proximal factors

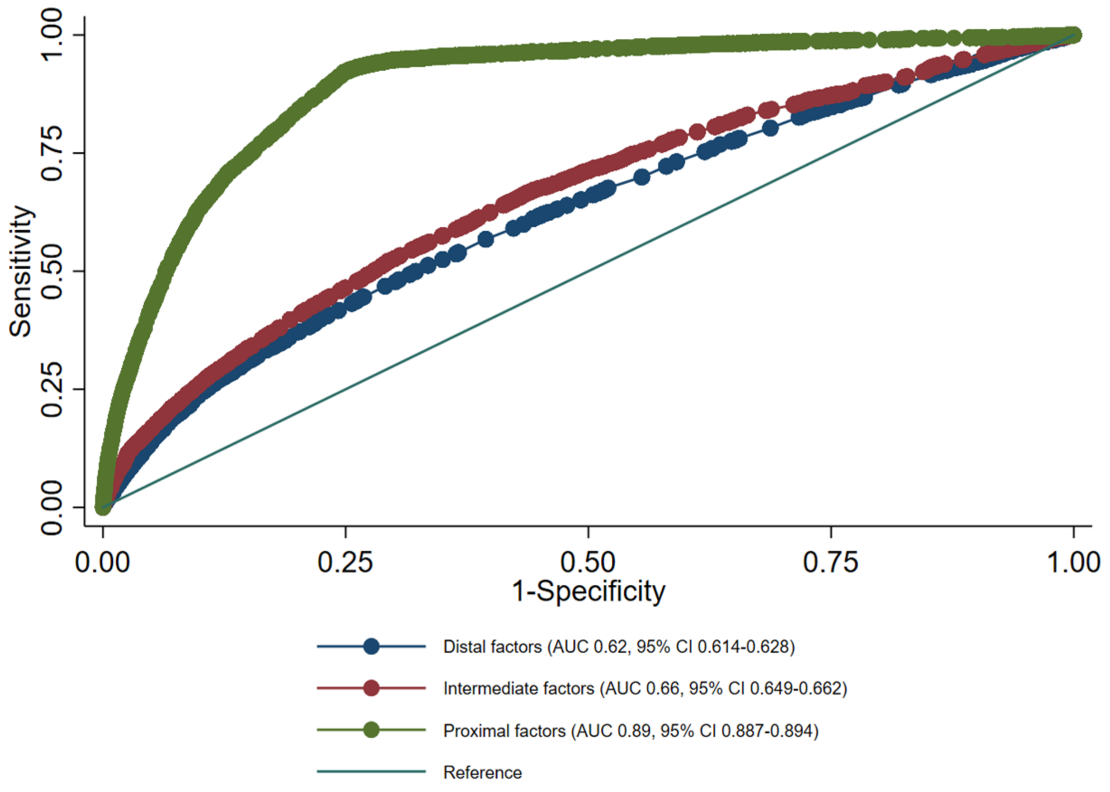

Note: Analysis was run using unweighted logistic regression.

Table-S1: Comparison of model estimates between unweighted and survey weighted data*

Variables	Unweighted model		Survey weighted model**	
	OR	*95% CI*	*OR*	*95% CI*
**Age**				
13-19	3.35	(3.05, 3.68)	3.66	(3.27, 4.10)
20-24	1.14	(1.07, 1.22)	1.16	(1.07, 1.26)
25-29	1.00	--	1.00	--
30-34	1.18	(1.09, 1.28)	1.19	(1.08, 1.31)
35-39	1.97	(1.81, 2.15)	1.92	(1.72, 2.13)
40-49	3.10	(2.82, 3.41)	2.80	(2.49, 3.15)
**Distal factors**				
**Place of residence**				
Urban	1.00	--	1.00	--
Rural	1.02	(0.94, 1.11)	0.97	(0.88, 1.07)
**Religion**				
Hindu	1.00	--	1.00	--
Buddhist	0.83	(0.21, 3.35)	0.58	(0.12, 2.77)
Christian	0.73	(0.60, 0.89)	0.58	(0.46, 0.74)
Jain	1.10	(0.41, 2.96)	1.16	(0.40, 3.36)
Muslim	0.66	(0.62, 0.72)	0.64	(0.59, 0.70)
Sikh	1.13	(0.66, 1.91)	1.08	(0.61, 1.92)
Other	0.77	(0.61, 0.97)	0.72	(0.54, 0.97)
**Social Group**				
Other	1.00	--	1.00	--
Scheduled caste	1.13	(1.06, 1.20)	1.10	(1.02, 1.18)
Scheduled tribe	1.52	(1.42, 1.64)	1.43	(1.31, 1.56)
**Wealth index**				
Wealthiest	1.00	--	1.00	--
Quintile 4	0.96	(0.89, 1.04)	0.99	(0.89, 1.09)
Quintile 3	0.96	(0.89, 1.04)	0.97	(0.88, 1.07)
Quintile 2	0.98	(0.91, 1.06)	0.97	(0.89, 1.07)
Lowest quintile	0.79	(0.73, 0.85)	0.81	(0.74, 0.90)
**Has health scheme**				
Yes	1.00	--	1.00	--
No	2.78	(2.52, 3.07)	2.72	(2.41, 3.07)
**Intermediate factors**				
**Accessed a health facility**				
Yes	1.00	--	1.00	--
No	2.93	(2.78, 3.09)	2.93	(2.75, 3.12)
**Proximal factors**				
**Pregnancy complication or medical comorbidity**				
No	1.00	--	1.00	--
Yes	48.6	(44.1, 53,7)	50.2	(44.5, 56.6)

*Estimates shown are for the final logistic regression model with adjustment for all distal, intermediate and proximal factors

**Survey weighted model is adjusted for clustering and survey design

For peer review only

Table – S2: Comparison of model estimates and precision using Taylor linearised standard errors and robust standard errors *

Variables	Taylor linearised**		Robust standard errors***	
	OR	*95% CI*	*OR*	*95% CI*
**Age**				
13-19	3.66	(3.27, 4.10)	3.66	(3.27, 4.10)
20-24	1.16	(1.07, 1.26)	1.16	(1.08, 1.26)
25-29	1.00	--	1.00	--
30-34	1.19	(1.08, 1.31)	1.19	(1.08, 1.31)
35-39	1.92	(1.72, 2.13)	1.92	(1.73, 2.13)
40-49	2.80	(2.49, 3.15)	2.80	(2.49, 3.15)
**Distal factors**				
**Place of residence**				
Urban	1.00	--	1.00	--
Rural	0.97	(0.88, 1.07)	0.97	(0.88, 1.07)
**Religion**				
Hindu	1.00	--	1.00	--
Buddhist	0.58	(0.12, 2.77)	0.58	(0.12, 2.77)
Christian	0.58	(0.46, 0.74)	0.58	(0.46, 0.74)
Jain	1.16	(0.40, 3.36)	1.17	(0.41, 3.36)
Muslim	0.64	(0.59, 0.70)	0.64	(0.59, 0.70)
Sikh	1.08	(0.61, 1.92)	1.08	(0.61, 1.92)
Other	0.72	(0.54, 0.97)	0.72	(0.54, 0.97)
**Social Group**				
Other	1.00	--	1.00	--
Scheduled caste	1.10	(1.02, 1.18)	1.10	(1.02, 1.18)
Scheduled tribe	1.43	(1.31, 1.56)	1.43	(1.31, 1.56)
**Wealth index**				
Wealthiest	1.00	--	1.00	--
Quintile 4	0.99	(0.89, 1.09)	0.99	(0.89, 1.09)
Quintile 3	0.97	(0.88, 1.07)	0.97	(0.88, 1.07)
Quintile 2	0.97	(0.89, 1.07)	0.97	(0.89, 1.07)
Lowest quintile	0.81	(0.74, 0.90)	0.81	(0.74, 0.90)
**Has health scheme**				
Yes	1.00	--	1.00	--
No	2.72	(2.41, 3.07)	2.72	(2.41, 3.07)
**Intermediate factors**				
**Accessed a health facility**				
Yes	1.00	--	1.00	--
No	2.93	(2.75, 3.12)	2.93	(2.75, 3.12)
**Proximal factors**				
**Pregnancy complication or medical comorbidity**				
No	1.00	--	1.00	--
Yes	50.2	(44.5, 56.6)	50.2	(44.5, 56.6)

*Estimates shown are for the final model with adjustment for all distal, intermediate and proximal factors
**The model using taylor linearised standard errors is estimating by adjustment for survey design using *svy* commands.
***The model using robust standard errors is estimated by adjustment for proportion weights.

For peer review only

Table – S3: Comparison of model estimates using complete-case analysis and multiple imputation by chained equations*

Variables	Imputed model**		Complete-case analysis***	
	OR	*95% CI*	*OR*	*95% CI*
**Age**				
13-19	3.68	(3.29, 4.13)	3.66	(3.27, 4.10)
20-24	1.17	(1.08, 1.27)	1.16	(1.07, 1.26)
25-29	1.00	--	1.00	--
30-34	1.18	(1.07, 1.29)	1.19	(1.08, 1.31)
35-39	1.86	(1.67, 2.06)	1.92	(1.72, 2.13)
40-49	2.70	(2.40, 3.03)	2.80	(2.49, 3.15)
**Distal factors**				
**Place of residence**				
Urban	1.00	--	1.00	--
Rural	0.98	(0.89, 1.07)	0.97	(0.88, 1.07)
**Religion**				
Hindu	1.00	--	1.00	--
Buddhist	0.55	(0.11, 2.61)	0.58	(0.12, 2.77)
Christian	0.64	(0.51, 0.80_	0.58	(0.46, 0.74)
Jain	1.17	(0.41, 3.36)	1.16	(0.40, 3.36)
Muslim	0.64	(0.59, 0.70)	0.64	(0.59, 0.70)
Sikh	1.09	(0.61, 1.93)	1.08	(0.61, 1.92)
Other	0.72	(0.53, 0.97)	0.72	(0.54, 0.97)
**Social Group**				
Other	1.00	--	1.00	--
Scheduled caste	1.09	(1.01, 1.17)	1.10	(1.02, 1.18)
Scheduled tribe	1.45	(1.33, 1.58)	1.43	(1.31, 1.56)
**Wealth index**				
Wealthiest	1.00	--	1.00	--
Quintile 4	0.99	(0.90, 1.09)	0.99	(0.89, 1.09)
Quintile 3	0.97	(0.88, 1.07)	0.97	(0.88, 1.07)
Quintile 2	0.97	(0.89, 1.07)	0.97	(0.89, 1.07)
Lowest quintile	0.83	(0.75, 0.91)	0.81	(0.74, 0.90)
**Has health scheme**				
Yes	1.00	--	1.00	--
No	2.68	(2.37, 3.02)	2.72	(2.41, 3.07)
**Intermediate factors**				
**Accessed a health facility**				
Yes	1.00	--	1.00	--
No	2.89	(2.72, 3.09)	2.93	(2.75, 3.12)
**Proximal factors**				
**Pregnancy complication or medical comorbidity**				
No	1.00	--	1.00	--
Yes	45.8	(41.0, 51.2)	50.2	(44.5, 56.6)

*Estimates shown are for the final model with adjustment for all distal, intermediate and proximal factors
** Multiple imputation was performed on the variable *accessing a health facility*. The imputed model uses proportion weighted data with robust standard errors
***The complete-case analysis model is survey weighted with Taylor linearised standard errors

For peer review only

Figure S1: Study population derivation

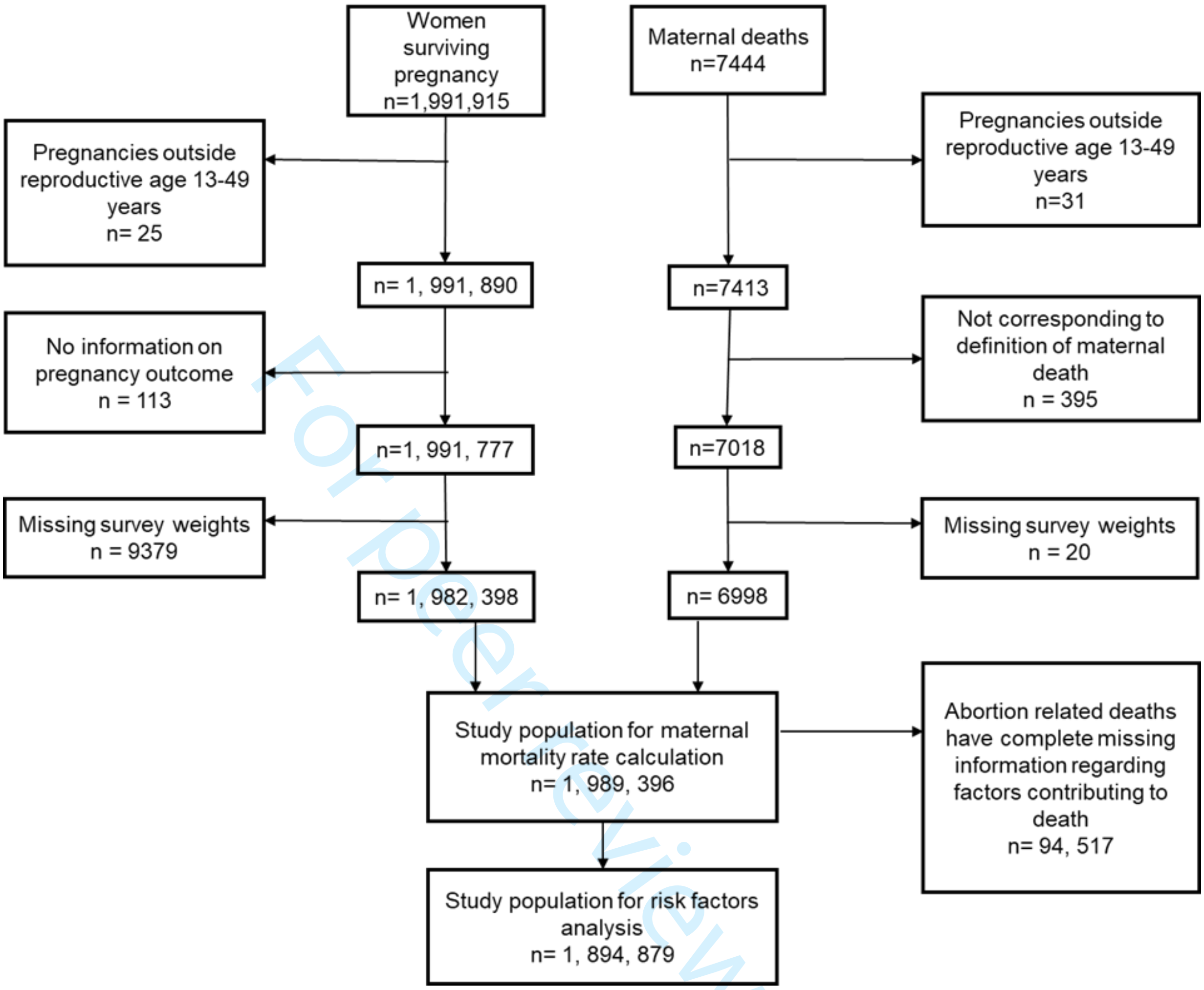

For peer review only

Figure S2: Theoretical framework for maternal mortality in India

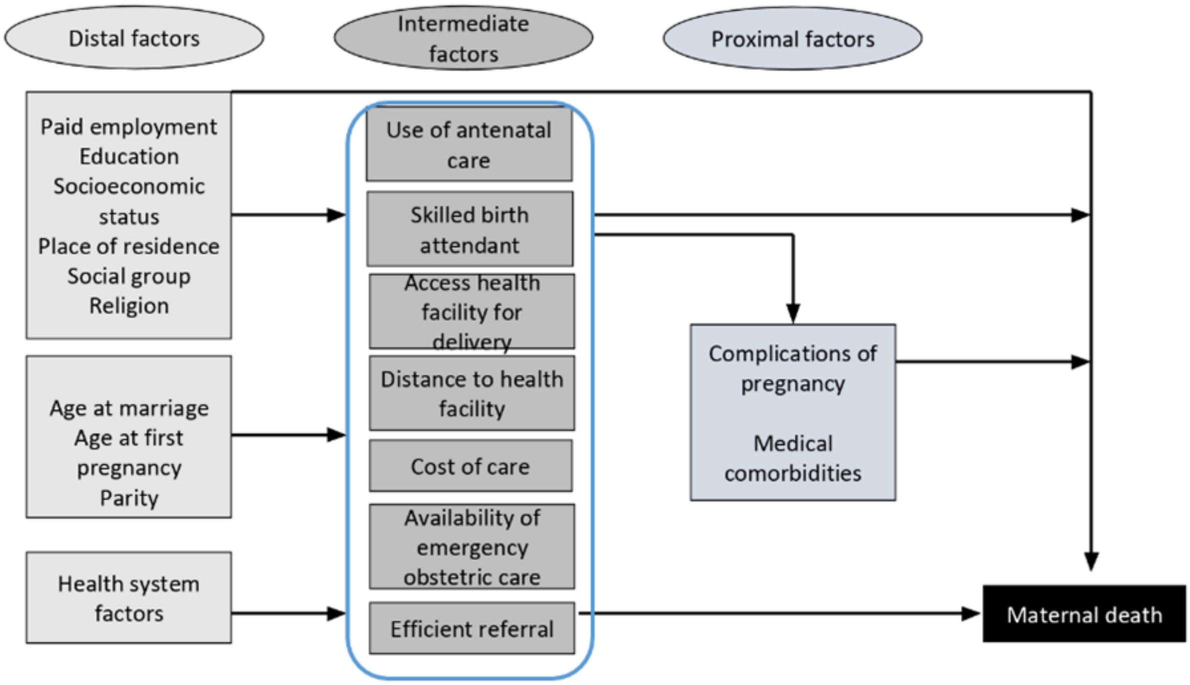

Figure S3: Odds ratios of maternal mortality according to maternal age with different levels of adjustment

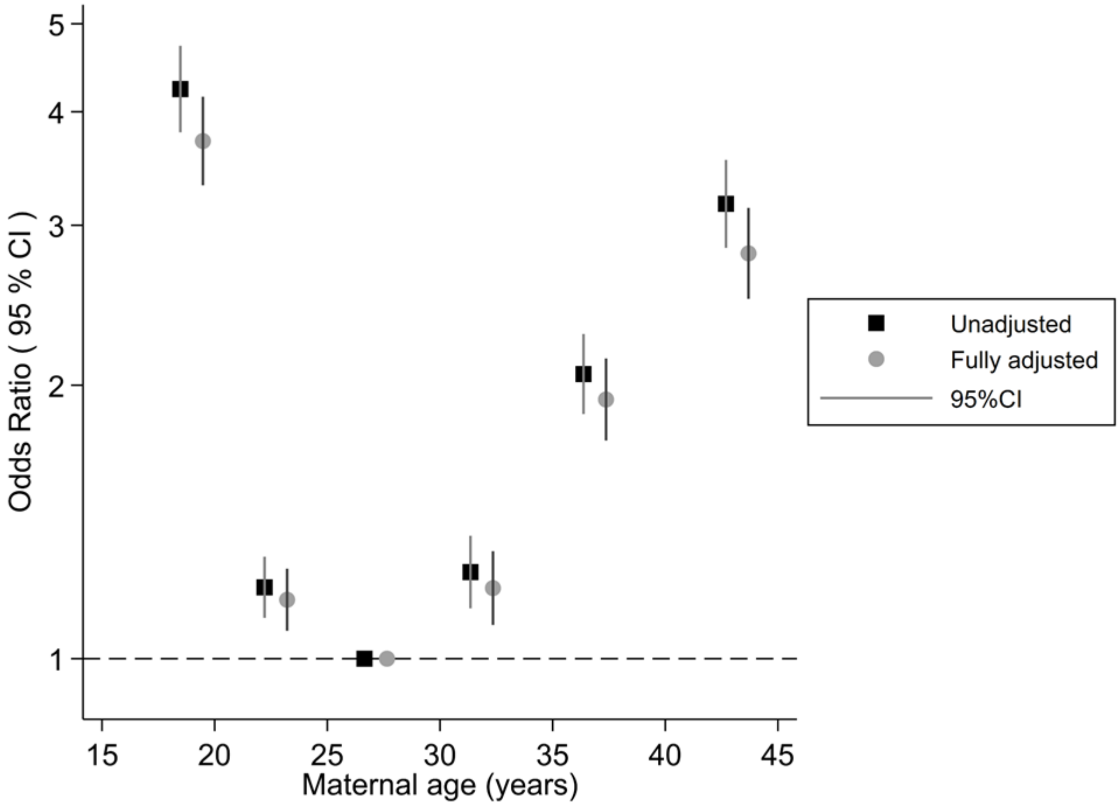

Note: Analyses were performed using logistic regression with adjustment for clustering and survey design. Odds ratios are shown with 95% confidence intervals. The fully adjusted model is fitted for all distal, intermediate and proximal factors.

Figure S4: Odds ratios of maternal death in rural vs. urban women with sequential adjustment

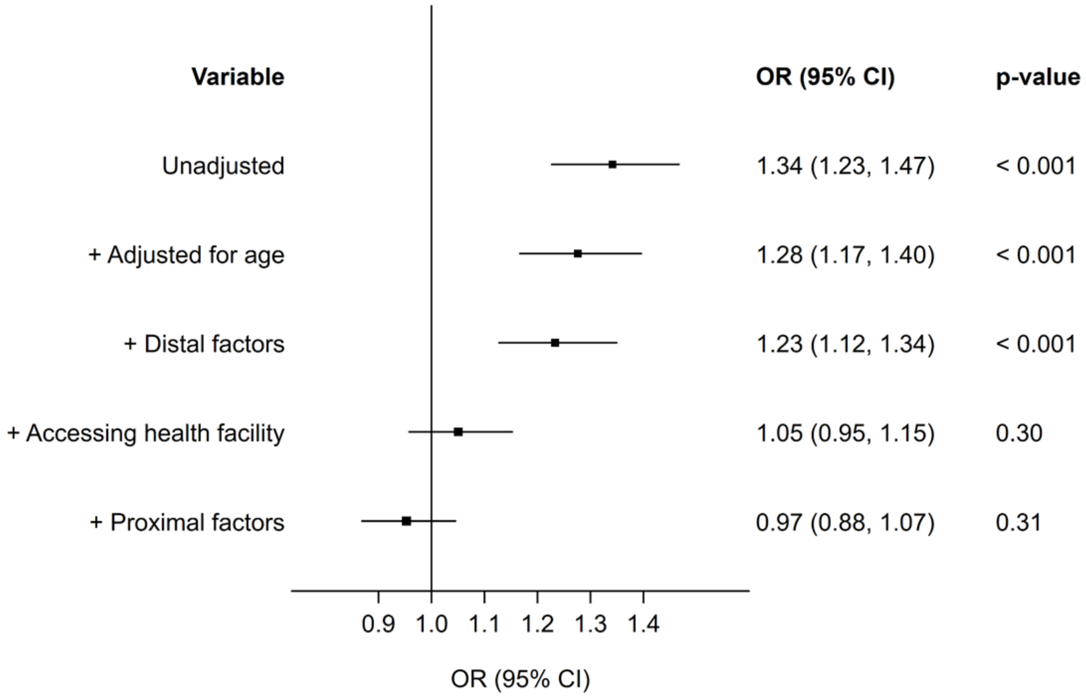

Note : Analyses were performed using logistic regression with adjustment for clustering and survey design. Size of data markers are proportional to the inverse of the variance of the odds ratio.

view only

Figure S5: Odds ratio of maternal death in poorest vs. wealthiest women with sequential adjustment

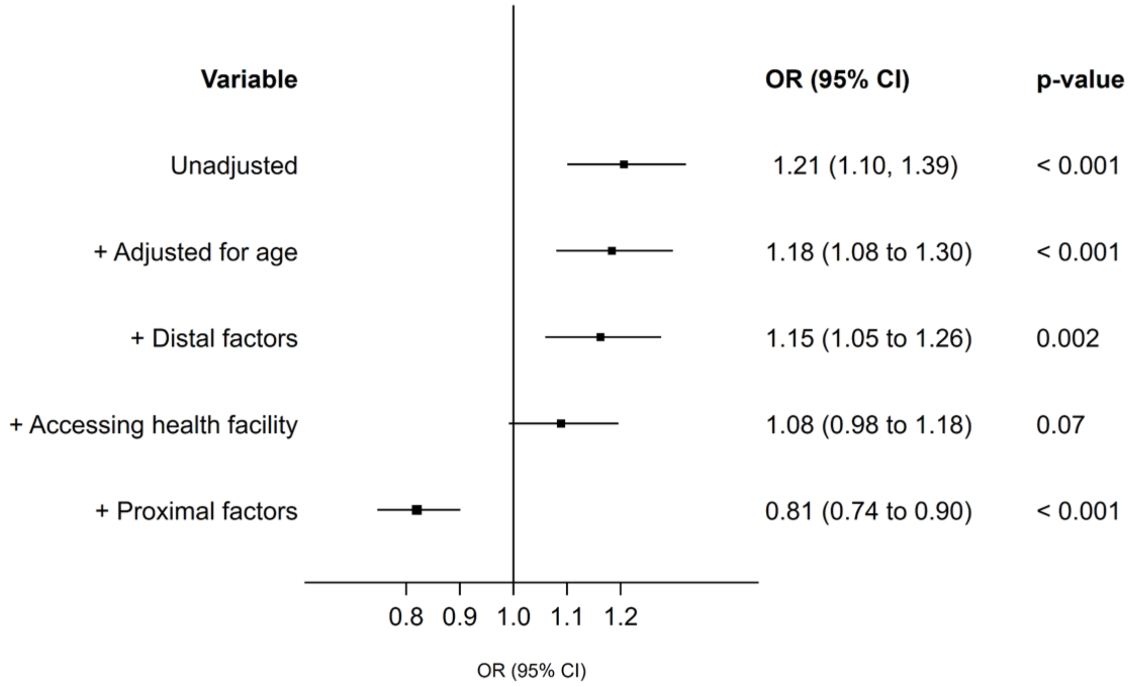

Note : Analyses were performed using logistic regression with adjustment for clustering and survey design. Size of data markers are proportional to the inverse of the variance of the odds ratio.

For peer review only

STROBE Statement—Checklist of items that should be included in reports of ***cross-sectional studies***

	Item No	Recommendation	Page No
**Title and abstract**	1	(*a*) Indicate the study's design with a commonly used term in the title or the abstract	1,2
		(*b*) Provide in the abstract an informative and balanced summary of what was done and what was found	2
**Introduction**			
Background/rationale	2	Explain the scientific background and rationale for the investigation being reported	4
Objectives	3	State specific objectives, including any prespecified hypotheses	4
**Methods**			
Study design	4	Present key elements of study design early in the paper	4
Setting	5	Describe the setting, locations, and relevant dates, including periods of recruitment, exposure, follow-up, and data collection	4,5
Participants	6	(*a*) Give the eligibility criteria, and the sources and methods of selection of participants	5
Variables	7	Clearly define all outcomes, exposures, predictors, potential confounders, and effect modifiers. Give diagnostic criteria, if applicable	4,5
Data sources/ measurement	8*	For each variable of interest, give sources of data and details of methods of assessment (measurement). Describe comparability of assessment methods if there is more than one group	5,6,7
Bias	9	Describe any efforts to address potential sources of bias	6,7
Study size	10	Explain how the study size was arrived at	5, Figure S1
Quantitative variables	11	Explain how quantitative variables were handled in the analyses. If applicable, describe which groupings were chosen and why	6,7, Table-1
Statistical methods	12	(*a*) Describe all statistical methods, including those used to control for confounding	6,7
		(*b*) Describe any methods used to examine subgroups and interactions	6,7
		(*c*) Explain how missing data were addressed	6,7
		(*d*) If applicable, describe analytical methods taking account of sampling strategy	6,7
		(*e*) Describe any sensitivity analyses	6,7
**Results**			
Participants	13*	(a) Report numbers of individuals at each stage of study—eg numbers potentially eligible, examined for eligibility, confirmed eligible, included in the study, completing follow-up, and analysed	5 and Figure-S1
		(b) Give reasons for non-participation at each stage	5 and Figure-S1
		(c) Consider use of a flow diagram	Figure-S1
Descriptive data	14*	(a) Give characteristics of study participants (eg demographic, clinical, social) and information on exposures and potential confounders	8,9, Table 1

For peer review only - http://bmjopen.bmj.com/site/about/guidelines.xhtml

		(b) Indicate number of participants with missing data for each variable of interest	Table 1
Outcome data	15*	Report numbers of outcome events or summary measures	5, Figure S1
Main results	16	(*a*) Give unadjusted estimates and, if applicable, confounder-adjusted estimates and their precision (eg, 95% confidence interval). Make clear which confounders were adjusted for and why they were included	Table-2
		(*b*) Report category boundaries when continuous variables were categorized	Table1, 2
		(*c*) If relevant, consider translating estimates of relative risk into absolute risk for a meaningful time period	Not applicable
Other analyses	17	Report other analyses done—eg analyses of subgroups and interactions, and sensitivity analyses	13, Figure 3
**Discussion**			
Key results	18	Summarise key results with reference to study objectives	13, 14
Limitations	19	Discuss limitations of the study, taking into account sources of potential bias or imprecision. Discuss both direction and magnitude of any potential bias	16,17
Interpretation	20	Give a cautious overall interpretation of results considering objectives, limitations, multiplicity of analyses, results from similar studies, and other relevant evidence	14,15,16,17
Generalisability	21	Discuss the generalisability (external validity) of the study results	15,16,17
**Other information**			
Funding	22	Give the source of funding and the role of the funders for the present study and, if applicable, for the original study on which the present article is based	19

*Give information separately for exposed and unexposed groups.

Note: An Explanation and Elaboration article discusses each checklist item and gives methodological background and published examples of transparent reporting. The STROBE checklist is best used in conjunction with this article (freely available on the Web sites of PLoS Medicine at http://www.plosmedicine.org/, Annals of Internal Medicine at http://www.annals.org/, and Epidemiology at http://www.epidem.com/). Information on the STROBE Initiative is available at www.strobe-statement.org.

For peer review only

For peer review only - http://bmjopen.bmj.com/site/about/guidelines.xhtml

BMJ Open

Risk factors for maternal mortality among 1.9 million women in nine Empowered Action Group states in India: secondary analysis of Annual Health Survey data

Journal:	*BMJ Open*
Manuscript ID	bmjopen-2020-038910.R1
Article Type:	Original research
Date Submitted by the Author:	11-Jun-2020
Complete List of Authors:	Horwood , Geneviève ; University of Oxford, Nuffield Department of Population Health<br>Opondo, Charles; University of Oxford, Nuffield Department of Population Health<br>Choudhury , Saswati; Gauhati Medical College and Hospital, Department of Obstetrics and Gynaecology<br>Rani, Anjali; Banaras Hindu University Institute of Medical Sciences, Department of Obstetrics and Gynaecology<br>Nair, Manisha; University of Oxford, NPEU, Nuffield Department of Population Health
**Primary Subject Heading**:	Epidemiology
Secondary Subject Heading:	Global health, Obstetrics and gynaecology
Keywords:	EPIDEMIOLOGY, OBSTETRICS, PUBLIC HEALTH

SCHOLARONE™
Manuscripts

BMJ

I, the Submitting Author has the right to grant and does grant on behalf of all authors of the Work (as defined in the below author licence), an exclusive licence and/or a non-exclusive licence for contributions from authors who are: i) UK Crown employees; ii) where BMJ has agreed a CC-BY licence shall apply, and/or iii) in accordance with the terms applicable for US Federal Government officers or employees acting as part of their official duties; on a worldwide, perpetual, irrevocable, royalty-free basis to BMJ Publishing Group Ltd ("BMJ") its licensees and where the relevant Journal is co-owned by BMJ to the co-owners of the Journal, to publish the Work in this journal and any other BMJ products and to exploit all rights, as set out in our licence.

The Submitting Author accepts and understands that any supply made under these terms is made by BMJ to the Submitting Author unless you are acting as an employee on behalf of your employer or a postgraduate student of an affiliated institution which is paying any applicable article publishing charge ("APC") for Open Access articles. Where the Submitting Author wishes to make the Work available on an Open Access basis (and intends to pay the relevant APC), the terms of reuse of such Open Access shall be governed by a Creative Commons licence – details of these licences and which Creative Commons licence will apply to this Work are set out in our licence referred to above.

Other than as permitted in any relevant BMJ Author's Self Archiving Policies, I confirm this Work has not been accepted for publication elsewhere, is not being considered for publication elsewhere and does not duplicate material already published. I confirm all authors consent to publication of this Work and authorise the granting of this licence.

For peer review only

Risk factors for maternal mortality among 1.9 million women in nine Empowered Action Group states in India: secondary analysis of Annual Health Survey data

Authors

1. Geneviève Horwood (MD, MSc)
   MSc student in Global Health Science, Nuffield Department of Population Health, University of Oxford
   Richard Doll Building, Old Road Campus, Headington, Oxford, OX3 7LF, UK
   Email: ghorwood@toh.ca

2. Charles Opondo (PhD)
   National Perinatal Epidemiology Unit, Nuffield Department of Population Health, University of Oxford
   Richard Doll Building, Old Road Campus, Headington, Oxford, OX3 7LF, UK
   Email: charles.opondo@npeu.ox.ac.uk

3. Saswati S Choudhury (MD)
   Department of Obstetrics and Gynaecology, GMCH, Guwahati
   Narkashur Hilltop, Christian Basti Bhangagarh, Guwahati, Assam 781032, India
   Email: saswatischoudhury@gmail.com

4. Anjali Rani (MD)
   Department of Obstetrics and Gynaecology, Institute of Medical Sciences, Banaras Hindu University,
   Ajagara, Banaras Hindu University Campus, Varanasi, Uttar Pradesh 221005, India
   Email: anjalirani.imsbhu@gmail.com

5. Manisha Nair (DPhil)
   National Perinatal Epidemiology Unit, Nuffield Department of Population Health, University of Oxford
   Richard Doll Building, Old Road Campus, Headington, Oxford, OX3 7LF, UK
   Email: manisha.nair@npeu.ox.ac.uk

Corresponding author

Manisha Nair
Senior Epidemiologist and Research Fellow,
National Perinatal Epidemiology Unit, NDPH, University of Oxford
Richard Doll Building, Old Road Campus, Headington, Oxford, OX3 7LF, UK
Email: manisha.nair@npeu.ox.ac.uk; Phone: +44-(0)1865-617820

For peer review only - http://bmjopen.bmj.com/site/about/guidelines.xhtml

ABSTRACT

Objective: To examine the risk factors for pregnancy-related death in India's nine Empowered Action Group States (EAG).

Design: Secondary data analysis of the Indian Annual Health Survey (2010-13)

Setting: Nine states: Assam, Bihar, Chhattisgarh, Jharkhand, Madhya Pradesh, Odisha, Rajasthan, Uttar Pradesh and Uttarakhand.

Participants: 1,989,396 pregnant women.

Methods: Maternal mortality ratio (MMR), overall and for each state, with 95% Confidence Interval (CI) were calculated. Stepwise multivariable logistic regression was used to investigate the association of risk factors with maternal mortality. Area under the receiver-operator characteristic curve (AUROC) was used to assess the prediction of the model.

Outcome measures: MMR adjusted for survey design, adjusted Odds Ratio (aOR) with 95% CI and C-statistic with 95% CI.

Results: MMR calculated for the nine states was 383/100,000 live births (95% CI 346 – 423 per 100,000). Age exhibited a U-shaped association with maternal mortality. Not having a health scheme and belonging to a scheduled caste or scheduled tribe group were significant risk factors for maternal death with aOR of 2.72 (95%CI:2.41-3.07), 1.10 (95%CI:1.02-1.18) and 1.43(95%CI:1.31-1.56), respectively. Socioeconomic status and rural residence were not associated with maternal mortality after adjusting for access to a healthcare facility. Complications of pregnancy and medical comorbidities were the strongest risk factors for maternal death (aOR 50.2, 95% CI:44.5-56.6). Together, the risk factors identified accounted for 89%(95%CI:0.887-0.894) of the AUROC.

Conclusions: Maternal mortality in India's EAG states greatly exceeds the national average. The identified risk factors demonstrate the importance of improving quality of pregnancy care. Notably, the study showed that the risk conferred by poor socioeconomic status could be mitigated by universal access to healthcare during pregnancy and childbirth.

ARTICLE SUMMARY

Strengths and limitations of this study

- This study identifies the risk factors for maternal mortality in nine socioeconomically disadvantaged states in India, a country with the second highest number of maternal deaths, globally.
- The study examines >7000 maternal deaths in a population of >1.9 million pregnant women, which makes this the large population-based study examining a wide range of risk factors for pregnancy related death in a vulnerable population in India where maternal death has been previously underreported.

For peer review only - http://bmjopen.bmj.com/site/about/guidelines.xhtml

- The focus of this study was on high burden disadvantaged states, thus the findings may not be generalizable to the rest of India.
- The survey design likely introduced non-systematic reporting bias leading to potential under-reporting of maternal deaths in early pregnancy and deaths due to indirect causes.

Keywords: Maternal death, India, risk factors, pregnancy

Word count: 3834

For peer review only

For peer review only - http://bmjopen.bmj.com/site/about/guidelines.xhtml

INTRODUCTION

Despite advances in modern medicine, pregnancy and childbirth remain one of the leading causes of mortality worldwide for women of reproductive age[1]. The question of safe motherhood is not merely one of public health concern but has been widely recognised as an issue of social injustice[2 3]. According to the Sustainable Development Goals, the global target is to reduce the maternal mortality ratio (MMR) to less than 70 per 100,000 live births by 2030 and to provide universal access to reproductive health care[4].

India is responsible for the second highest number of maternal deaths worldwide(5). India's maternal mortality ratio has been steadily declining since the 1990s[1 5 6]. According to the Sample Registration System, a household survey conducted by the Indian government, the MMR dropped from over 400 per 100, 000 in the early 1990s, to 230 in 2008, and to 130 per 100,000 between 2014 and 2016[5]. In comparison, the global MMR in 2015 was estimated at 216 (95% Confidence Interval (CI) 207 to 249)[6]. However, this downward trend masks staggering within-country variations in maternal mortality.. For example, in the southern state of Kerala, in India, the MMR was reported to be as low as 61 per 100,000 live births in 2013 whereas in the northern state of Bihar it was 208 per 100,000 live births[5]. This inequality in the burden of maternal deaths between northern and southern states is reflected by other socio-economic indicators such as poverty and education[7] which could influence access to care during pregnancy and postpartum[8].

The government of India launched the National Rural Health Mission (NRHM) in 2005, to improve access to good quality healthcare services for socioeconomically disadvantaged population concentrating its efforts on nine Empowered Action Group States (EAG)[9 10]. These are Assam, Bihar, Chhattisgarh, Jharkhand, Madhya Pradesh, Odisha, Rajasthan, Uttar Pradesh and Uttarakhand. The EAG states and Assam together account for about 50% of the total population, 61% of the total births, 71% of the infant deaths, and 72% of the Under-5 deaths in India[10]. Under NRHM, multiple maternal health programmes were developed. Examples include the Janani

For peer review only - http://bmjopen.bmj.com/site/about/guidelines.xhtml

Suraksha Yojana (JSY), a cash-incentive scheme[11], whose objective is to promote institutional deliveries, and introduction of a large cadre of community health workers known as ASHAs (Accredited Social Health Activists). These health schemes are generally aimed at impoverished or marginalised women to facilitate access to health care.

Despite these efforts, little is known about the population level risk factors that influence maternal death in the vulnerable population in these states. The objective of this study was to examine the risk factors for pregnancy-related death in India's nine EAG states using a large-scale population-based dataset.

METHODS

Study design

This study is a secondary data analysis of the Annual Health Survey (AHS), a government household survey conducted between 2010 and 2013 in the nine EAG states.

Data Source

The Annual Health Survey (AHS) is a large household survey of over 4 million households. The survey was conducted every year between 2010 and 2013 in the nine EAG states. Compared with the Demographic and Household surveys, AHS collected more detailed information on events and outcomes related to women's reproductive health and pregnancy occurring between the 1st January 2007 and the 31st of December 2009[10]. The survey used a stratified simple random sampling strategy. It recorded information regarding demographic and socioeconomic characteristics, reproductive and sexual health information, and maternal death. The maternal death questionnaire gathered information from a member of the household in which a woman had died in pregnancy or postpartum during the reference period[10]. The questionnaire focused on identified delays to care and factors contributing to death, but the methodology was not comparable to the conventional verbal autopsy. The information on 'delay' in the dataset were mainly categorised into 'delay on the part of the woman or the family in recognising the danger

For peer review only - http://bmjopen.bmj.com/site/about/guidelines.xhtml

signs or seriousness of the complication', 'delay in accessing healthcare', or 'delay in receiving appropriate healthcare'.

Study Sample

The study included 1,991,915 women reporting a pregnancy within the defined survey time frame and 7444 pregnancy-related deaths as reported by a member of the same household. Women were excluded if their age was outside the reproductive window. While the WHO considers the reproductive age to be 15-49 years, pregnancies occurring at age 13 and above were considered plausible and were included in the analysis. Women with missing survey weights or missing pregnancy outcomes were excluded. In the mortality data set, deaths occurring outside the time frame included in the definition of maternal death (see box 1) were excluded. While abortion-related deaths were recorded, the survey did not ask household members to identify factors associated with death in the case of women dying after an abortion. Therefore, all women, whether survived or died (including abortion-related deaths) were included in calculating the MMRs, but due to the lack of information, women who had an abortion in the reported index pregnancy (whether survived or died) were excluded from the risk factor analysis. Figure S1 (Supplementary file) depicts the study sample derivation.

Box 1: Definitions related to maternal death

Maternal death: Death of a woman while pregnant or within 42 days of termination of pregnancy, irrespective of the duration and site of the pregnancy, from any cause related to or aggravated by the pregnancy or its management, but not from accidental or incidental causes[5].

Maternal mortality ratio: Number of maternal deaths per 100,000 livebirths[5].

Variable choice and extraction

For peer review only - http://bmjopen.bmj.com/site/about/guidelines.xhtml

A systematic search of the literature was undertaken by interrogating the Medline, Embase and Global Health databases as well as the WHO's list of publications, to identify potential risk factors for maternal death in India. Using the literature search, hypothesized predictors of maternal mortality specific to our study setting were divided into broad categories of distal, intermediate and proximal factors guided by the WHO's conceptual framework of the social determinants of health[12]. The theoretical framework is depicted in Figure S2.

This theoretical framework guided the choice of variables used in the statistical analysis. The mortality questionnaire was brief, so few of the variables identified by the literature review were available. Of the distal factors identified in the framework, those available were place of residence, wealth index, having a health scheme *(defined as whether the family received benefits from JSY, government health insurance and other health and financial benefit schemes)*, religion and social group *(categorised as 'Schedule caste (SC)', 'Schedule tribe (ST)' and 'Others'; SC and ST are considered socioeconomically disadvantaged groups)*. Of the intermediate factors, the only variable available was accessing a health facility. For the proximal factors, a binary variable was created distinguishing between women with any complication of pregnancy or medical comorbidity and women with neither. Comorbidities included hypertensive disorders of pregnancy, sepsis, bleeding, jaundice, obstructed or prolonged labour, other pregnancy complications, and other medical conditions. Women surviving pregnancy were classified as accessing a health facility if they delivered in a health facility. Women dying in pregnancy were classified as accessing a health facility if they died in a health facility. This classification was based on the presumption that both active labour and acute complications eventual leading to death were comparably crucial events in pregnancy requiring urgent medical care. The questionnaire addressed to surviving women had gathered further information regarding access to care in pregnancy including antenatal care and birth attendant. Conversely, the maternal death questionnaire only included place of death. Both groups' interaction with the health care system was therefore solely comparable based on place of death or delivery.

For peer review only - http://bmjopen.bmj.com/site/about/guidelines.xhtml

Statistical analysis

Frequency distributions of binary and categorical variables as well as measures of central tendency for continuous variables were reported. Baseline characteristics were assessed and contrasted between maternal deaths and all surviving women. Frequency distributions of maternal deaths by identified delays to care and by time period of death in relation to pregnancy were examined, stratified by registered and unregistered deaths *(defined based on whether the death was registered with the local registrar for vital statistics within the administrative unit)*. The maternal mortality ratio (defined in Box-1) with 95% CI was calculated in each state and for the region overall using survey weighted counts of maternal deaths and live births.

The univariable association of each potential risk factor with maternal death was explored using simple logistic regression. Stepwise logistic regression was then used to build a multivariable model. The sequential addition of variables to the model was informed by the theoretical framework, where distal factors were added first, followed by intermediate and proximal factors. Statistical interaction between social group and wealth quintiles was tested. To inform appropriate modelling of the effect of age, tests for departure from linearity and trend were conducted. If the effects of risk factors were found to be markedly attenuated after full adjustment, they were explored further by assessing confounding of the association by distal, intermediate, and proximal factors.

Observations were not expected to be independent within clusters, therefore analyses were adjusted for clustering and sampling design. Wald test estimation-values were reported and a modified Hosmer-Lemeshow test, appropriate for clustered data, was used to assess model goodness of fit[13]. To assess the potential effect of clustering on the variance of the model estimates, standard errors were compared between models using Taylor linearised estimates of the variance and models using robust standard errors. In addition, point estimates and

For peer review only - http://bmjopen.bmj.com/site/about/guidelines.xhtml

confidence intervals were compared between models with adjustment for clustering and those without adjustment.

Patterns and amounts of missing data were described for each of the included variables in the final model. While missing information could have been related to other independent variables, it was not thought to be associated with the outcome, and therefore, the data were presumed to be missing at random. An indicator variable for missingness was created for each variable with missing data to explore reasons for missingness using logistic regression. Since the outcome variable was binary (died or survived), multiple imputation by chained equations (MICE) was used as a method of choice for imputing missing data, and models containing imputed datasets were compared to the complete-case analysis model. As the variables included in our analysis had very small proportion of missing data, complete-case analysis was retained as our final model.

To assess the risk prediction of each of the groups of risk factors (distal, intermediate, and proximal), the areas under the receiver-operator characteristic (AUROC) curves for the models corresponding to the addition of each of these groups were compared.

All analyses were carried out using Stata 14.2. Two-tailed p-values of less than 0.05 were considered statistically significant.

Patient and Public Involvement

Patient and public were not involved in this secondary analysis of anonymous survey data**.**

RESULTS

Table 1 shows the baseline characteristics of women included in the study. The median age was 26 years and was the same for women surviving and women dying in pregnancy. In general, the households with maternal deaths were more likely to be from a rural area, to be from the two lower wealth quintiles, to belong to a scheduled caste or scheduled tribe social group and to not have a health scheme. Of the women surviving pregnancy, 53.1% accessed a health facility compared to only 36.1% of women dying in pregnancy. In addition, 92.4% of the women dying

For peer review only - http://bmjopen.bmj.com/site/about/guidelines.xhtml

in pregnancy suffered at least one complication of pregnancy, compared to 26.3% of women surviving pregnancy.

Maternal mortality ratio

The overall maternal mortality ratio was 383 per 100,000 live births (95% CI 346 – 423 per 100,000). Two states had an MMR of over 500: Assam (552, 95% CI 507 - 600 per 100,000) and Odisha (534, 95% CI 490 – 581 per 100,000).. The state with the lowest MMR was Uttarakhand accounting for 209 maternal deaths per 100,000 live births (95% CI 182 – 239 per 100,000). No state had an MMR below 200 (Figure 1).

Delays contributing to maternal death and time-period of death

Figure 2 depicts maternal death according to the identified delay most contributing to the death. The delay most frequently cited was inappropriate quality of care at the health facility (55.1%). Lack of care seeking was the second most important delay (18.1%), followed by difficulty in reaching the facility (15.3%). In the groups expressing a delay in care seeking or in reaching the facility, over 80% had not registered the death. Conversely, in those expressing a delay in quality of care, 57% had registered the death. The majority of the deaths were in the antenatal period (about 40%), followed by intrapartum and postpartum deaths. More than half of the deaths in each time period were unregistered (Figure 3).

For peer review only

For peer review only - http://bmjopen.bmj.com/site/about/guidelines.xhtml

Table 1: Baseline characteristics of the women included in the study population*		
	**Surviving women**	**Maternal deaths**
**N**	1982398	6998
**Age (yrs)****	26.0 (23.0, 30.0)	26.0 (22.0, 32.0)
**State**		
Assam	168 040 (5%)	812 (7.1%)
Bihar	353 043 (18.5%)	1247 (18.6%)
Chhattisgarh	106 416 (4.1%)	335 (3.5%)
Jharkhand	162 987 (5.3%)	647 (4.8%)
Madhya Pradesh	233 964 (12.0%)	742 (11.2%)
Odisha	121 777 (4.7%)	649 (6.5%)
Rajasthan	150 668 (9.1%)	529 (8.9%)
Uttar Pradesh	538 160 (39.9%)	1778 (38.6%)
Uttarakhand	147 343 (1.5%)	259 (0.8%)
**Region of residence**		
Rural	1 690 156 (82.8%)	6118 (85.5%)
Urban	292 242 (17.2%)	738 (13.1%)
Missing§	0	142 (1.5%)
**Religion**		
Buddhist	956 (<0.01%)	2 (<0.01%)
Christian	26 109 (1.1%)	114 (1.2%)
Hindu	1 632 888 (81.4%)	5813 (83.0%)
Jain	2074 (0.1%)	4 (<0.01%)
Muslim	292 640 (16.4%)	903 (13.9%)
Sikh	4713 (0.2%)	14 (0.2%)
Other	21 206 (0.5%)	83 (0.6%)
Missing§	1812 (0.1%)	65 (1.1 %)
**Social group**		
Scheduled caste	386 169 (20.6%)	1533 (23.3%)
Scheduled tribe	244 057 (10.5%)	1082 (12.2%)
Others	1 350 426 (68.7%)	4318 (63.4%)
Missing§	1746 (0.1%)	65 (1.1%)
**Has health scheme**		
No	1 826 156 (92.7%)	6552 (93.9%)
Yes	15 6242 (7.3%)	446 (6.1%)
**Wealth index quintile**		
Poorest	397 828 (20.1%)	1492 (21.9%)
2	397 338 (21.8%)	1588 (23.9%)
3	397 600 (20.4%)	1400 (20.6%)
4	395 890 (18.7%)	1269 (17.5%)
Wealthiest	393 742 (18.3%)	1249 (16.1%)
**Accessing a health facility**		
Yes	1 066 136 (53.1%)	2579 (36.1%)
No	798 811 (40.1%)	4322 (63.0%)
Missing§	117 451 (6.01%)	97 (0.9%)
**Pregnancy complication or comorbidity**		
No	1 460 887 (73.0%)	535 (6.6%)
Yes	521 511 (27.0%)	6463 (93.4%)

* Characteristics are presented as unweighted counts and survey weighted frequencies. ** Age is presented as the median with interquartile range. §Missing reported here includes women with abortion-related deaths who were not included in the final model for risk factor analysis.

For peer review only - http://bmjopen.bmj.com/site/about/guidelines.xhtml

Risk factors for maternal mortality

There was a departure from linearity in the association between age and maternal death and as such, age was coded as a categorical variable. Women between the ages of 25 to 29 years were least likely to die during pregnancy. The youngest age group, women between 13 and 19 years, was the group with the highest risk of death in pregnancy (adjusted odds ratio (aOR) 3.66, 95% CI 3.27-4.10), followed by the eldest group of women of between 40 and 49 years of age (aOR 2.80, 95% CI 2.49-3.15). Age exhibited a strong u-shaped relationship with maternal death and this relationship was largely unchanged after full adjustment for other risk factors (Figure S3).

After adjustment for all distal factors, the effect size of the association between rural residence, social group, health scheme, and maternal death were all mildly attenuated but remained significant. There was a trend of association across wealth quintile groups and maternal death. Associations between not having a health scheme, accessing a health facility or the presence of a complication with maternal death were all strengthened after adjustment for distal factors (Table 2). However, attenuation of the associations of place of residence, wealth index and social group with maternal death occurred after adjustment for the presence of a complication or medical comorbidity (Table 2). Being of a scheduled caste or scheduled tribe group continued to be significantly associated with maternal death. Not having a health scheme conferred significant risk (aOR 2.72, 95%CI 2.41-3.07) after adjustment for other factors.

After taking into account the access to a health facility, the association between rural residence and maternal death was attenuated (aOR 1.05, 95% CI 0.95-1.15). In addition, the trend of association between wealth quintile groups and maternal death was no longer significant and there was no evidence of association between wealth index and dying in pregnancy. Figures S4 and S5 depict the attenuation of the association between maternal mortality and both wealth and rural residence with sequential adjustment for other risk factors.

For peer review only - http://bmjopen.bmj.com/site/about/guidelines.xhtml

Table 2: Risk factors for maternal mortality with progressive adjustment										
**Variables**	**Unadjusted**		**Adjusted for age**		**Distal factors***		**Intermediate factors†**		**Proximal factors‡**	
	OR	***95% CI***	***OR***	***95% CI***	***OR***	***95% CI***	***OR***	***95% CI***	***OR***	***95% CI***
**Age (years)**										
13-19	4.23	(3.80, 4.73)	NA	--	4.12	(3.69, 4.62)	4.34	(3.87, 4.85)	3.66	(3.27, 4.10)
20-24	1.20	(1.11, 1.30)	NA	--	1.20	(1.11, 1.30)	1.25	(1.15, 1.35)	1.16	(1.07, 1.26)
25-29	1.00	--	NA	--	1.00	--	1.00	--	1.00	--
30-34	1.25	(1.14, 1.37)	NA	--	1.25	(1.14, 1.37)	1.20	(1.10, 1.32)	1.19	(1.08, 1.31)
35-39	2.06	(1.86, 2.27)	NA	--	2.03	(1.83, 2.25)	1.91	(1.72, 2.12)	1.92	(1.72, 2.13)
40-49	3.17	(2.83, 3.54)	NA	--	3.13	(2.79, 3.51)	2.91	(2.60, 3.27)	2.80	(2.49, 3.15)
**Distal factors**										
**Place of residence**										
Urban	1.00	--	1.00	--	1.00	--	1.00	--	1.00	--
Rural	1.34	(1.23, 1.47)	1.28	(1.17, 1.40)	1.23	(1.12, 1.34)	1.05	(0.95, 1.15)	0.97	(0.88, 1.07)
**Religion**										
Hindu	1.00	--	1.00	--	1.00	--	1.00	--	1.00	--
Buddhist	0.43	(0.09, 2.01)	0.45	(0.10, 2.12)	0.46	(0.10, 2.14)	0.52	(0.11, 2.46)	0.58	(0.12, 2.77)
Christian	1.03	(0.82, 1.30)	1.01	(0.80, 1.26)	1.02	(0.81, 1.28)	0.90	(0.70, 1.14)	0.58	(0.46, 0.74)
Jain	0.58	(0.20, 1.65)	0.63	(0.22, 1.79)	0.77	(0.27, 2.20)	0.91	(0.32, 2.61)	1.16	(0.40, 3.36)
Muslim	0.83	(0.76, 0.91)	0.79	(0.73, 0.86)	0.84	(0.77, 0.92)	0.76	(0.69, 0.83)	0.64	(0.59, 0.70)
Sikh	0.86	(0.48, 1.52)	0.94	(0.53, 1.67)	1.03	(0.58, 1.82)	1.12	(0.63, 1.99)	1.08	(0.61, 1.92)
Other	1.03	(0.78, 1.37)	0.96	(0.72, 1.27)	0.88	(0.66, 1.18)	0.76	(0.56, 1.02)	0.72	(0.54, 0.97)
**Social Group**										
Other	1.00	--	1.00	--	1.00	--	1.00	--	1.00	--
Scheduled caste	1.22	(1.14, 1.31)	1.20	(1.12, 1.29)	1.15	(1.06, 1.23)	1.10	(1.02, 1.19)	1.10	(1.02, 1.18)
Scheduled tribe	1.24	(1.14, 1.34)	1.19	(1.10, 1.29)	1.15	(1.05, 1.26)	1.11	(1.02, 1.21)	1.43	(1.31, 1.56)
**Wealth index**										
Wealthiest	1.00	--	1.00	--	1.00	--	1.00	--	1.00	--
Quintile 4	1.07	(0.97, 1.17)	1.07	(0.97, 1.17)	1.07	(0.97, 1.18)	1.03	(0.94, 1.14)	0.99	(0.89, 1.09)
Quintile 3	1.16	(1.06, 1.27)	1.14	(1.04, 1.25)	1.17	(1.06, 1.28)	1.09	(0.99, 1.20)	0.97	(0.88, 1.07)
Quintile 2	1.25	(1.14, 1.37)	1.21	(1.11, 1.32)	1.22	(1.11, 1.33)	1.11	(1.02, 1.22)	0.97	(0.89, 1.07)
Lowest quintile	1.21	(1.10, 1.32)	1.18	(1.08, 1.30)	1.15	(1.05, 1.26)	1.08	(0.98, 1.18)	0.81	(0.74, 0.90)
**Has health scheme**										
Yes	1.00	--	1.00	--	1.00	--	1.00	--	1.00	--
No	1.24	(1.10, 1.39)	1.26	(1.12, 1.41)	1.36	(1.21, 1.53)	1.33	(1.18, 1.50)	2.72	(2.41, 3.07)
**Intermediate factors**										
**Accessed health facility**										
Yes	1.00	--	1.00	--	1.00	--	NA	--	1.00	--
No	2.26	(2.13, 2.40)	2.21	(2.09, 2.35)	2.22	(2.08, 2.36)	NA	--	--	2.93
**Proximal factors****										
No	1.00	--	1.00	--	1.00	--	1.00	--	1.00	--
Yes	36.5	(32.8, 40.7)	36.2	(32.6, 40.3)	40.1	(35.9, 44.8)	50.2	(44.5, 56.6)	NA	--

For peer review only - http://bmjopen.bmj.com/site/about/guidelines.xhtml

§ All analyses use logistic regression and are adjusted for clustering and survey design; * Further adjusted for distal factors †Further adjusted for accessing health facility ‡Further adjusted for proximal factors; ** Pregnancy complication or medical comorbidity

For peer review only

For peer review only - http://bmjopen.bmj.com/site/about/guidelines.xhtml

The magnitude of the association between proximal factors (pregnancy complications and medical comorbidities) and maternal death was further increased after adjustment for the distal and intermediate factors (aOR 50.2, 95% CI 44.5-56.6).

F-statistics adjusted for survey design as a measure of contribution to the model fit showed that the variables accounting for the greatest amount of variation in the data were proximal factors, accessing a health facility, not having a health scheme, and age. Model diagnostics showed no evidence of collinearity between dependent variables or model misspecification.

In assessing clustering of the data, standard errors were compared between models with survey weighted data using Taylor linearization variance estimates and proportion weighted data using robust standard errors. These were found to be equivalent. Point estimates and standard errors were similar between models with unweighted data and survey weighted data. These findings suggested that clustering effects were minimal in the study population. Further, the findings were comparable in imputed models and complete-case analysis with a marginal widening of the 95% CIs. See Tables S1, S2 and S3 in the supplementary file.

Figure 4 shows the area under the receiver-operator characteristic curve for each group of risk factors. Together, the distal and intermediate factors accounted for 66% (95% CI 0.649-0.662) of the variance in the data. With the addition of the proximal factors, this increased to 89% (95% CI 0.887-0.894).

DISCUSSION

Our study showed that the maternal mortality ratio in the nine states was much higher than the corresponding WHO estimates for India's overall MMR for a similar time-period. Lack of quality care in the health facilities were perceived as the factor most contributing to the maternal deaths by family members of deceased women. Proximal factors, which encompassed any pregnancy complication or medical comorbidity, were the strongest predictors of women dying in pregnancy followed by lack of access to health facility. More importantly, the study showed that the effects

For peer review only - http://bmjopen.bmj.com/site/about/guidelines.xhtml

For peer review only

of living rurally and being poor could be mitigated by improving access to health facility. Other risk factors identified were younger or older maternal age, belonging to a scheduled tribe or caste social group, and not being enrolled in a health scheme.

The WHO reported MMR for India in 2010 was 208/100,000 live births[1 6] compared with 383/100,000 live births calculated in our study population for a similar period (2007-2009). This discrepancy reflects the high burden of maternal mortality in India's nine socioeconomically disadvantaged states. Moreover, in our study, more than half of the maternal deaths had not been previously recorded. Efforts to create a national electronic database for maternal death surveillance in India began in 2013[14], but the progress is still limited, mainly due to lack of resources[15 16].

Our study identified two key modifiable health systems determinants of maternal mortality in the nine disadvantaged states – access to health facility and quality of care. Research in many low-and-middle-income countries (LMICs) consistently show that living in rural areas and being poor are associated with decreased odds of childbirth in health facility, antenatal care, and skilled attendance at birth[17 18]. Our study demonstrated that the effects of wealth and living rurally were mediated by a woman's ability to access care and do not exert a direct effect on maternal mortality. This is an important finding in the context of India and other LMICs in terms of universal access to antenatal, delivery and postnatal care. Access to appropriate good quality care could mitigate the effects of socioeconomic inequalities know to be associated with a high burden of maternal mortality.

The AUROC curve analysis provides evidence that proximal factors (pregnancy complications and medical co-morbidities) were the strongest predictors of maternal mortality in this population. Further, the association between proximal factors and maternal death was strengthened after accounting for other factors showing that these complications remain the most important risk factors for maternal death, thus reinforcing the importance of high quality

For peer review only - http://bmjopen.bmj.com/site/about/guidelines.xhtml

antenatal care, availability of basic emergency obstetrical care, and postnatal care. Nonetheless, in our study population, poor quality of care was reported as the factor most contributing to the maternal death in 55% of the deaths.

In 2005, the Indian government implemented the National Rural Heath Mission to address gaps in maternal health care. Improving the access to and quality of care, particularly for rural areas in high focus states, were the main objectives of this initiative[9]. Since 2005, India has seen some improvements. Institutional deliveries increased from 39% in 2005 to 79% in 2013. Complete antenatal care coverage increased from 37% to 51% and postnatal care increased from 27% to 36%[19]. However, studies show that the programme has had little or no effect on clinical outcomes. For example, despite the increase in health facility deliveries, there has been no demonstrated decline in maternal mortality[11 20].

Studies have repeatedly shown that quality of care is lacking in public health facilities including non-availability of essential medicines such as uterotonics and antibiotics, and lack of facilities for caesarean section and blood transfusion[21]. Further, government reports on the state of public facilities in these nine states have reported lack of toilet facilities, unreliable electricity, and unsatisfactory cleanliness[22], and lack of basic measures of obstetrical care such as adequate hand hygiene[23]. Moreover, there have been reports of verbal and physical abuse of staff towards patients[8 22].

Conforming with the findings of other studies[24 25], age and maternal death exhibited a strong U-shaped association in which the youngest (women aged 13 to 19 years) and the oldest age group (women aged 40-49 years) had the highest risk of dying. India's National Family Health Survey showed that of women aged 20 to 49, 27% had been married before the age of 15 and 58% were married before the legal age of marriage of 18 years. In addition, 30% of Indian women had given birth by age 19[19]. While age itself is not a modifiable risk factor, childbearing-age should be regarded as one. Women should be able to exert control over when they choose

For peer review only - http://bmjopen.bmj.com/site/about/guidelines.xhtml

to become pregnant. Policies supporting universal access to contraception, family planning counselling, safe abortion, and promoting women's empowerment need continued attention in India.

This is a large population-based study conducted using a survey with minimal sampling bias and included a sample size of almost two million women and over 7000 recorded maternal deaths. The AHS database had two advantages over other household surveys in India: (i) it had more detailed information about a range of potential risk factors that we set out to examine in this study, and (ii) it allowed us to focus our analysis on socioeconomically disadvantaged states with a high burden of maternal deaths. We were able to study a population where maternal deaths are often unreported and where vital statistics and health related data are lacking. One limitation is that the AHS was conducted in 2010-13 and it is possible that the risk factors identified in our study may have changed over the years. However, they are unlikely to have changed significantly considering that these nine states continue to have a higher maternal mortality rate than the rest of India. The risk factors included in the regression model were informed by both established theory in the field of maternal health and a current review of the literature specific to the study setting, and the analysis addressed distal, intermediate, and proximal causes of maternal death crucial to the understanding of women's health dynamics in the most vulnerable population in India's EAG states.

We acknowledge that information collected about women dying in pregnancy was limited and did not include several factors identified as important by the literature review such as education, paid employment, parity, age at first pregnancy, distance to the health facility or having a skilled birth attendant. However, a majority of these factors would have been covered by the 'wealth index' and 'access to health facility'. The survey design is also likely to have introduced non-systematic reporting bias. In keeping with previous literature, underreporting of early pregnancy deaths, particularly those arising from complications of abortion or deaths prior to detecting pregnancy, and of maternal deaths arising from indirect causes that might have been reported as deaths due

For peer review only - http://bmjopen.bmj.com/site/about/guidelines.xhtml

For peer review only

to medical causes (ignoring the pregnancy state) could have led to an underestimation of maternal deaths[26].

Maternal mortality in India's EAG states greatly exceeds the national average and is far from the target set out by the Sustainable Development Goals (SDGs). Analysis of the results in the context of India's health care system suggests that targeted interventions to improve maternal outcomes in this population would include improved in-hospital care, improved access to healthcare services, and guaranteed skilled personnel and basic emergency obstetrical care in every maternity centre. Reducing maternal mortality in India's socioeconomically disadvantaged states is achievable but will require continued accelerated national efforts to recognise the importance of women's lives and invest in sustainable health systems founded on principles of accountability and evidence-based medicine.

REFERENCES

1. Alkema L, Chou D, Hogan D, et al. Global, regional, and national levels and trends in maternal mortality between 1990 and 2015, with scenario-based projections to 2030: a systematic analysis by the UN Maternal Mortality Estimation Inter-Agency Group. *Lancet (London, England)* 2016;387(10017):462-74. doi: 10.1016/s0140-6736(15)00838-7 [published Online First: 2015/11/21]
2. Rosenfield A, Maine D. Maternal mortality--a neglected tragedy. Where is the M in MCH? *Lancet (London, England)* 1985;2(8446):83-5. doi: 10.1016/s0140-6736(85)90188-6 [published Online First: 1985/07/13]
3. WHO. Reduction of Maternal Mortality: A Joint WHO/UNFPA/UNICEF World Bank Statement. Geneva: WHO, 1999.
4. World Health Organisation. MDG 5:Improve maternal health Geneva: WHO; 2015 [cited 2020 07 February]. Available from: https://www.who.int/topics/millennium_development_goals/maternal_health/en/ accessed 07 February 2020.
5. Registrar General of India Sample registration system. Special Bulletin on Maternal Mortality in India 2014-16 Sample Registration System. *SRS Bulletin* 2018(91):16–8.
6. WHO. Trends in maternal mortality: 1990 to 2015. Geneva: WHO, 2018.
7. The World Bank Group. Kerala: Indicators at a glance: The World Bank Group, 2017.
8. Nair H, Panda R. Quality of maternal healthcare in India: Has the National Rural Health Mission made a difference? *J Glob Health* 2011;1(1):79-86.
9. Chatterjee P. The health system in India: the underserved majority. *Lancet (London, England)* 2017;390(10111):2426-27. doi: 10.1016/s0140-6736(17)32860-x [published Online First: 2017/11/19]
10. Office of the Registrar General & Census Commissioner. Annual Health Survey Report: A report on Core and Vital Health Indicators- Part 1, 2016.

For peer review only - http://bmjopen.bmj.com/site/about/guidelines.xhtml

11. Randive B, Diwan V, De Costa A. India's Conditional Cash Transfer Programme (the JSY) to Promote Institutional Birth: Is There an Association between Institutional Birth Proportion and Maternal Mortality? *PLoS One* 2013;8(6):e67452. doi: 10.1371/journal.pone.0067452 [published Online First: 2013/07/05]
12. WHO. Closing the gap : policy into practice on social determinants of health : discussion paper. Geneva: WHO, 2011.
13. Archer KJ, Lemeshow S, Hosmer DW. Goodness-of-fit tests for logistic regression models when data are collected using a complex sampling design. *Computational Statistics & Data Analysis* 2007;51(9):4450-64. doi: https://doi.org/10.1016/j.csda.2006.07.006
14. Purandare C, Bhardwaj A, Malhotra M, et al. Every death counts: Electronic tracking systems for maternal death review in India. *International Journal of Gynecology & Obstetrics* 2014;127:S35-S39. doi: https://doi.org/10.1016/j.ijgo.2014.09.003
15. Gupta M, Rao C, Lakshmi PVM, et al. Estimating mortality using data from civil registration: a cross-sectional study in India. *Bull World Health Organ* 2016;94(1):10-21. doi: 10.2471/BLT.15.153585 [published Online First: 11/02]
16. Rane TM, Mahanta TG, Islam S, et al. Civil registration system (CRS) for birth and death registration in Assam – A rapid assessment. *Clinical Epidemiology and Global Health* 2019 doi: https://doi.org/10.1016/j.cegh.2019.05.006
17. Ahmed S, Creanga AA, Gillespie DG, et al. Economic Status, Education and Empowerment: Implications for Maternal Health Service Utilization in Developing Countries. *PLOS ONE* 2010;5(6):e11190. doi: 10.1371/journal.pone.0011190
18. Bauserman M, Lokangaka A, Thorsten V, et al. Risk factors for maternal death and trends in maternal mortality in low- and middle-income countries: a prospective longitudinal cohort analysis. *Reproductive health* 2015;12 Suppl 2:S5. doi: 10.1186/1742-4755-12-s2-s5 [published Online First: 2015/06/13]
19. International Institute for Population Sciences. National Family Health Survey (NFHS-4) 2015-16 India *International Institute for Population Sciences (IIPS) and ICF* 2017:1–192. doi: http://rchiips.org/NFHS/NFHS-4Reports/India.pdf
20. Lim SS, Dandona L, Hoisington JA, et al. India's Janani Suraksha Yojana, a conditional cash transfer programme to increase births in health facilities: an impact evaluation. *The Lancet* 2010;375(9730):2009-23. doi: 10.1016/S0140-6736(10)60744-1
21. Chaturvedi S, Randive B, Diwan V, et al. Quality of obstetric referral services in India's JSY cash transfer programme for institutional births: a study from Madhya Pradesh province. *PLoS One* 2014;9(5):e96773. doi: 10.1371/journal.pone.0096773 [published Online First: 2014/05/09]
22. Ministry of Health & Family Welfare Government of India. Second Common Review Mission Report, 2008:1–124.
23. Spector JM, Lashoher A, Agrawal P, et al. Designing the WHO Safe Childbirth Checklist program to improve quality of care at childbirth. *International journal of gynaecology and obstetrics: the official organ of the International Federation of Gynaecology and Obstetrics* 2013;122(2):164-8. doi: 10.1016/j.ijgo.2013.03.022 [published Online First: 2013/06/08]
24. Blanc AK, Winfrey W, Ross J. New Findings for Maternal Mortality Age Patterns: Aggregated Results for 38 Countries. *PLOS ONE* 2013;8(4):e59864. doi: 10.1371/journal.pone.0059864
25. Nove A, Matthews Z, Neal S, et al. Maternal mortality in adolescents compared with women of other ages: evidence from 144 countries. *The Lancet Global Health* 2014;2(3):e155-e64. doi: 10.1016/S2214-109X(13)70179-7
26. Yokoe R, Rowe R, Choudhury SS, et al. Unsafe abortion and abortion-related death among 1.8 million women in India. *BMJ Global Health* 2019;4(3):e001491. doi: 10.1136/bmjgh-2019-001491

For peer review only - http://bmjopen.bmj.com/site/about/guidelines.xhtml

Conflicts of interest: The authors declare that we have no competing interests.

Author statement: GH reviewed the literature, conducted the analysis and wrote the first draft of the paper; CO supervised the data analysis, interpretation and discussion of the results, and edited the paper; SSC helped in acquiring and interpreting the data, and edited the paper; AR edited the paper; MN developed the concept for the study, supervised the data analysis, interpretation and discussion of the results, and edited the paper.

Ethical approval: Ethics approval was not required since this was a secondary analysis of anonymous survey data.

Funding: The study was funded by a Medical Research Council Career Development Award to Manisha Nair (Grant Ref: MR/P022030/1). The funder had no role in the study design, data analysis, data interpretation, or writing of the manuscript. All authors, had full access to all of the data in the study and can take responsibility for the integrity of the data and the accuracy of the data analysis.

Data statement
The anonymised data is freely available through the Indian Government's Data Sharing Portal.

Figure legends

Figure 1: Maternal mortality ratio by state and overall

Figure 2: Frequencies of maternal death according to the delay cited by household members as most contributing to the death

Figure 3: Frequencies of maternal death according to the time period in relation to pregnancy

Figure 4: Area under the receiver-operator characteristic curve for distal, intermediate and proximal factors

For peer review only - http://bmjopen.bmj.com/site/about/guidelines.xhtml

Figure-1: Maternal mortality ratio (MMR) by state and overall

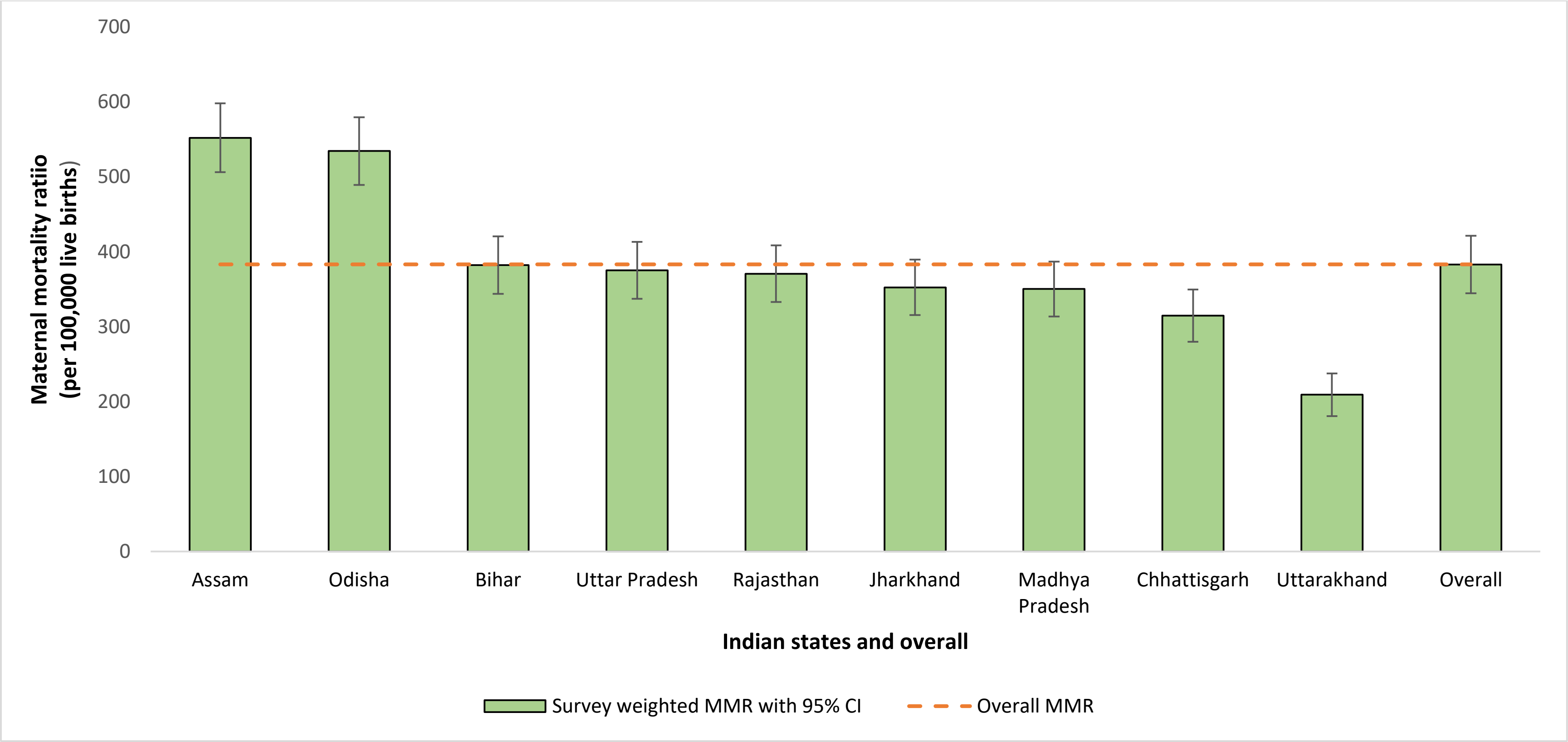

Source of data: Annual Health Survey, India

Figure 2: **Frequencies of maternal death according to the delay cited by household members as most contributing to the death**

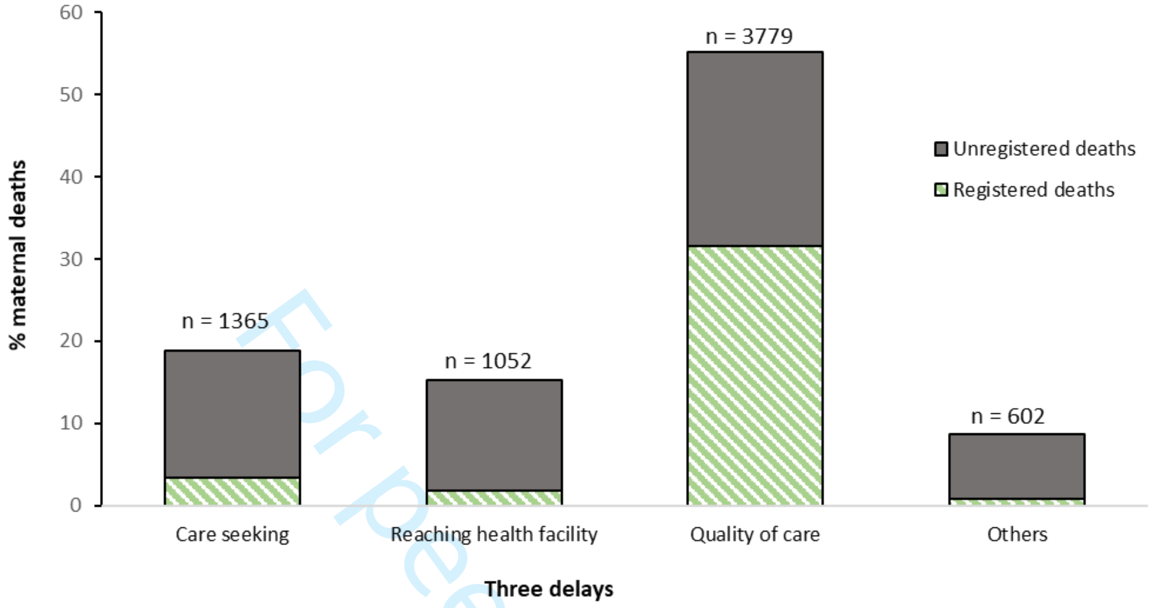

Note: Maternal death frequencies are presented as survey weighted frequencies. Unweighted counts of maternal deaths for each delay are presented over each bar. For each delay, maternal deaths are divided by proportion of registered and unregistered deaths.

For peer review only

Figure 3: Frequencies of maternal death according to the time period in relation to pregnancy

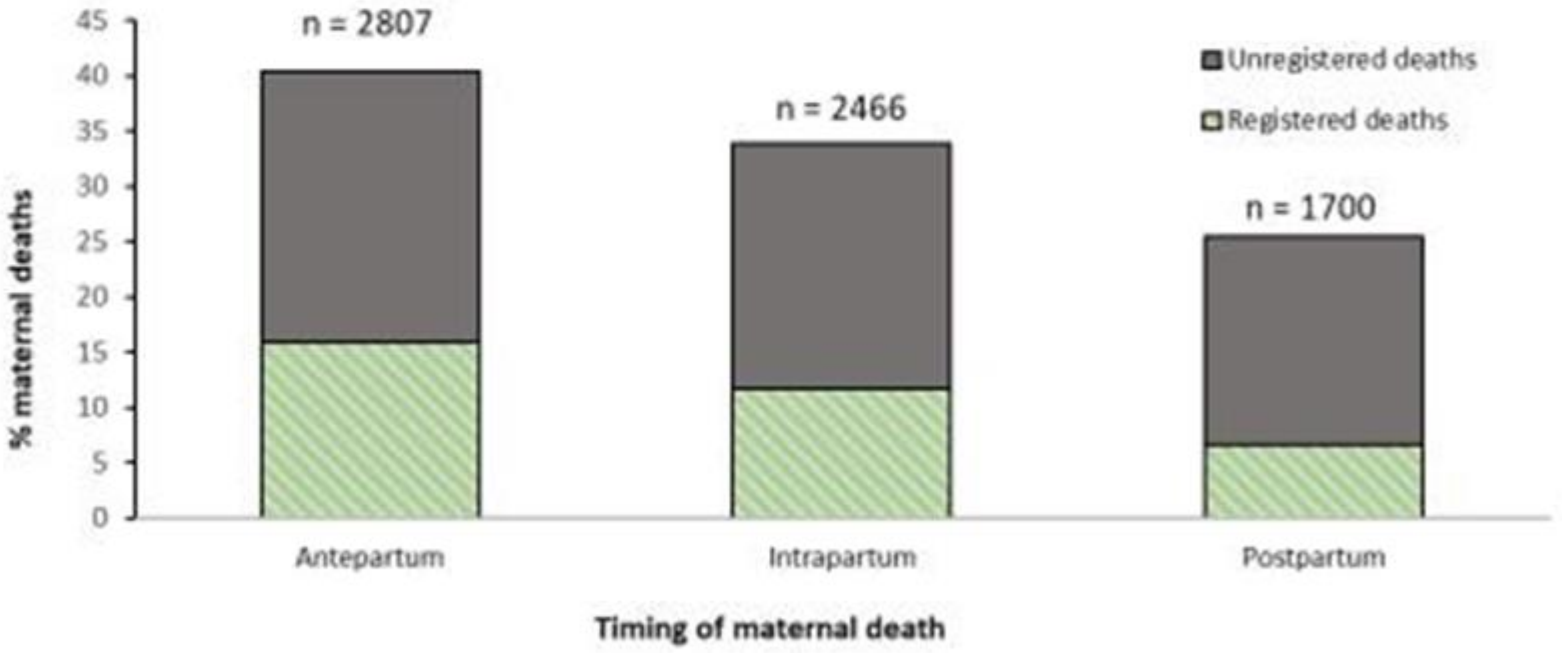

Note: Maternal death frequencies are presented as survey weighted frequencies. Unweighted counts of maternal deaths for each time period are presented over each bar. In each time period, maternal deaths are divided by proportion of registered and unregistered deaths.

Figure 4: Area under the receiver-operator characteristic curve for distal, intermediate and proximal factors

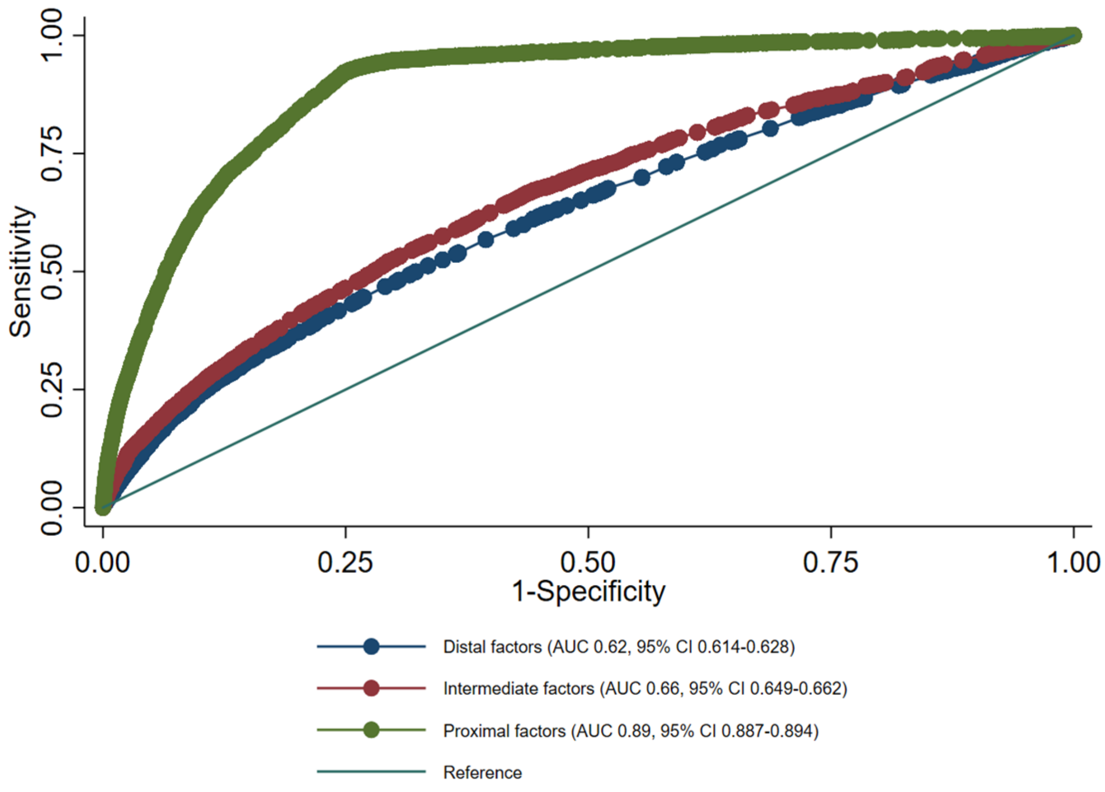

Note: Analysis was run using unweighted logistic regression.

For peer review only - http://bmjopen.bmj.com/site/about/guidelines.xhtml

Table-S1: Comparison of model estimates between unweighted and survey weighted data*

Variables	Unweighted model		Survey weighted model**	
	OR	*95% CI*	*OR*	*95% CI*
**Age**				
13-19	3.35	(3.05, 3.68)	3.66	(3.27, 4.10)
20-24	1.14	(1.07, 1.22)	1.16	(1.07, 1.26)
25-29	1.00	--	1.00	--
30-34	1.18	(1.09, 1.28)	1.19	(1.08, 1.31)
35-39	1.97	(1.81, 2.15)	1.92	(1.72, 2.13)
40-49	3.10	(2.82, 3.41)	2.80	(2.49, 3.15)
**Distal factors**				
**Place of residence**				
Urban	1.00	--	1.00	--
Rural	1.02	(0.94, 1.11)	0.97	(0.88, 1.07)
**Religion**				
Hindu	1.00	--	1.00	--
Buddhist	0.83	(0.21, 3.35)	0.58	(0.12, 2.77)
Christian	0.73	(0.60, 0.89)	0.58	(0.46, 0.74)
Jain	1.10	(0.41, 2.96)	1.16	(0.40, 3.36)
Muslim	0.66	(0.62, 0.72)	0.64	(0.59, 0.70)
Sikh	1.13	(0.66, 1.91)	1.08	(0.61, 1.92)
Other	0.77	(0.61, 0.97)	0.72	(0.54, 0.97)
**Social Group**				
Other	1.00	--	1.00	--
Scheduled caste	1.13	(1.06, 1.20)	1.10	(1.02, 1.18)
Scheduled tribe	1.52	(1.42, 1.64)	1.43	(1.31, 1.56)
**Wealth index**				
Wealthiest	1.00	--	1.00	--
Quintile 4	0.96	(0.89, 1.04)	0.99	(0.89, 1.09)
Quintile 3	0.96	(0.89, 1.04)	0.97	(0.88, 1.07)
Quintile 2	0.98	(0.91, 1.06)	0.97	(0.89, 1.07)
Lowest quintile	0.79	(0.73, 0.85)	0.81	(0.74, 0.90)
**Has health scheme**				
Yes	1.00	--	1.00	--
No	2.78	(2.52, 3.07)	2.72	(2.41, 3.07)
**Intermediate factors**				
**Accessed a health facility**				
Yes	1.00	--	1.00	--
No	2.93	(2.78, 3.09)	2.93	(2.75, 3.12)
**Proximal factors**				
**Pregnancy complication or medical comorbidity**				
No	1.00	--	1.00	--
Yes	48.6	(44.1, 53,7)	50.2	(44.5, 56.6)

*Estimates shown are for the final logistic regression model with adjustment for all distal, intermediate and proximal factors

**Survey weighted model is adjusted for clustering and survey design

For peer review only

Table – S2: Comparison of model estimates and precision using Taylor linearised standard errors and robust standard errors *

Variables	Taylor linearised**		Robust standard errors***	
	OR	*95% CI*	*OR*	*95% CI*
**Age**				
13-19	3.66	(3.27, 4.10)	3.66	(3.27, 4.10)
20-24	1.16	(1.07, 1.26)	1.16	(1.08, 1.26)
25-29	1.00	--	1.00	--
30-34	1.19	(1.08, 1.31)	1.19	(1.08, 1.31)
35-39	1.92	(1.72, 2.13)	1.92	(1.73, 2.13)
40-49	2.80	(2.49, 3.15)	2.80	(2.49, 3.15)
**Distal factors**				
**Place of residence**				
Urban	1.00	--	1.00	--
Rural	0.97	(0.88, 1.07)	0.97	(0.88, 1.07)
**Religion**				
Hindu	1.00	--	1.00	--
Buddhist	0.58	(0.12, 2.77)	0.58	(0.12, 2.77)
Christian	0.58	(0.46, 0.74)	0.58	(0.46, 0.74)
Jain	1.16	(0.40, 3.36)	1.17	(0.41, 3.36)
Muslim	0.64	(0.59, 0.70)	0.64	(0.59, 0.70)
Sikh	1.08	(0.61, 1.92)	1.08	(0.61, 1.92)
Other	0.72	(0.54, 0.97)	0.72	(0.54, 0.97)
**Social Group**				
Other	1.00	--	1.00	--
Scheduled caste	1.10	(1.02, 1.18)	1.10	(1.02, 1.18)
Scheduled tribe	1.43	(1.31, 1.56)	1.43	(1.31, 1.56)
**Wealth index**				
Wealthiest	1.00	--	1.00	--
Quintile 4	0.99	(0.89, 1.09)	0.99	(0.89, 1.09)
Quintile 3	0.97	(0.88, 1.07)	0.97	(0.88, 1.07)
Quintile 2	0.97	(0.89, 1.07)	0.97	(0.89, 1.07)
Lowest quintile	0.81	(0.74, 0.90)	0.81	(0.74, 0.90)
**Has health scheme**				
Yes	1.00	--	1.00	--
No	2.72	(2.41, 3.07)	2.72	(2.41, 3.07)
**Intermediate factors**				
**Accessed a health facility**				
Yes	1.00	--	1.00	--
No	2.93	(2.75, 3.12)	2.93	(2.75, 3.12)
**Proximal factors**				
**Pregnancy complication or medical comorbidity**				
No	1.00	--	1.00	--
Yes	50.2	(44.5, 56.6)	50.2	(44.5, 56.6)

*Estimates shown are for the final model with adjustment for all distal, intermediate and proximal factors
**The model using taylor linearised standard errors is estimating by adjustment for survey design using *svy* commands.
***The model using robust standard errors is estimated by adjustment for proportion weights.

For peer review only

Table – S3: Comparison of model estimates using complete-case analysis and multiple imputation by chained equations*

Variables	Imputed model**		Complete-case analysis***	
	OR	*95% CI*	*OR*	*95% CI*
**Age**				
13-19	3.68	(3.29, 4.13)	3.66	(3.27, 4.10)
20-24	1.17	(1.08, 1.27)	1.16	(1.07, 1.26)
25-29	1.00	--	1.00	--
30-34	1.18	(1.07, 1.29)	1.19	(1.08, 1.31)
35-39	1.86	(1.67, 2.06)	1.92	(1.72, 2.13)
40-49	2.70	(2.40, 3.03)	2.80	(2.49, 3.15)
**Distal factors**				
**Place of residence**				
Urban	1.00	--	1.00	--
Rural	0.98	(0.89, 1.07)	0.97	(0.88, 1.07)
**Religion**				
Hindu	1.00	--	1.00	--
Buddhist	0.55	(0.11, 2.61)	0.58	(0.12, 2.77)
Christian	0.64	(0.51, 0.80_	0.58	(0.46, 0.74)
Jain	1.17	(0.41, 3.36)	1.16	(0.40, 3.36)
Muslim	0.64	(0.59, 0.70)	0.64	(0.59, 0.70)
Sikh	1.09	(0.61, 1.93)	1.08	(0.61, 1.92)
Other	0.72	(0.53, 0.97)	0.72	(0.54, 0.97)
**Social Group**				
Other	1.00	--	1.00	--
Scheduled caste	1.09	(1.01, 1.17)	1.10	(1.02, 1.18)
Scheduled tribe	1.45	(1.33, 1.58)	1.43	(1.31, 1.56)
**Wealth index**				
Wealthiest	1.00	--	1.00	--
Quintile 4	0.99	(0.90, 1.09)	0.99	(0.89, 1.09)
Quintile 3	0.97	(0.88, 1.07)	0.97	(0.88, 1.07)
Quintile 2	0.97	(0.89, 1.07)	0.97	(0.89, 1.07)
Lowest quintile	0.83	(0.75, 0.91)	0.81	(0.74, 0.90)
**Has health scheme**				
Yes	1.00	--	1.00	--
No	2.68	(2.37, 3.02)	2.72	(2.41, 3.07)
**Intermediate factors**				
**Accessed a health facility**				
Yes	1.00	--	1.00	--
No	2.89	(2.72, 3.09)	2.93	(2.75, 3.12)
**Proximal factors**				
**Pregnancy complication or medical comorbidity**				
No	1.00	--	1.00	--
Yes	45.8	(41.0, 51.2)	50.2	(44.5, 56.6)

*Estimates shown are for the final model with adjustment for all distal, intermediate and proximal factors
** Multiple imputation was performed on the variable *accessing a health facility*. The imputed model uses proportion weighted data with robust standard errors
***The complete-case analysis model is survey weighted with Taylor linearised standard errors

For peer review only

Figure S1: Study population derivation

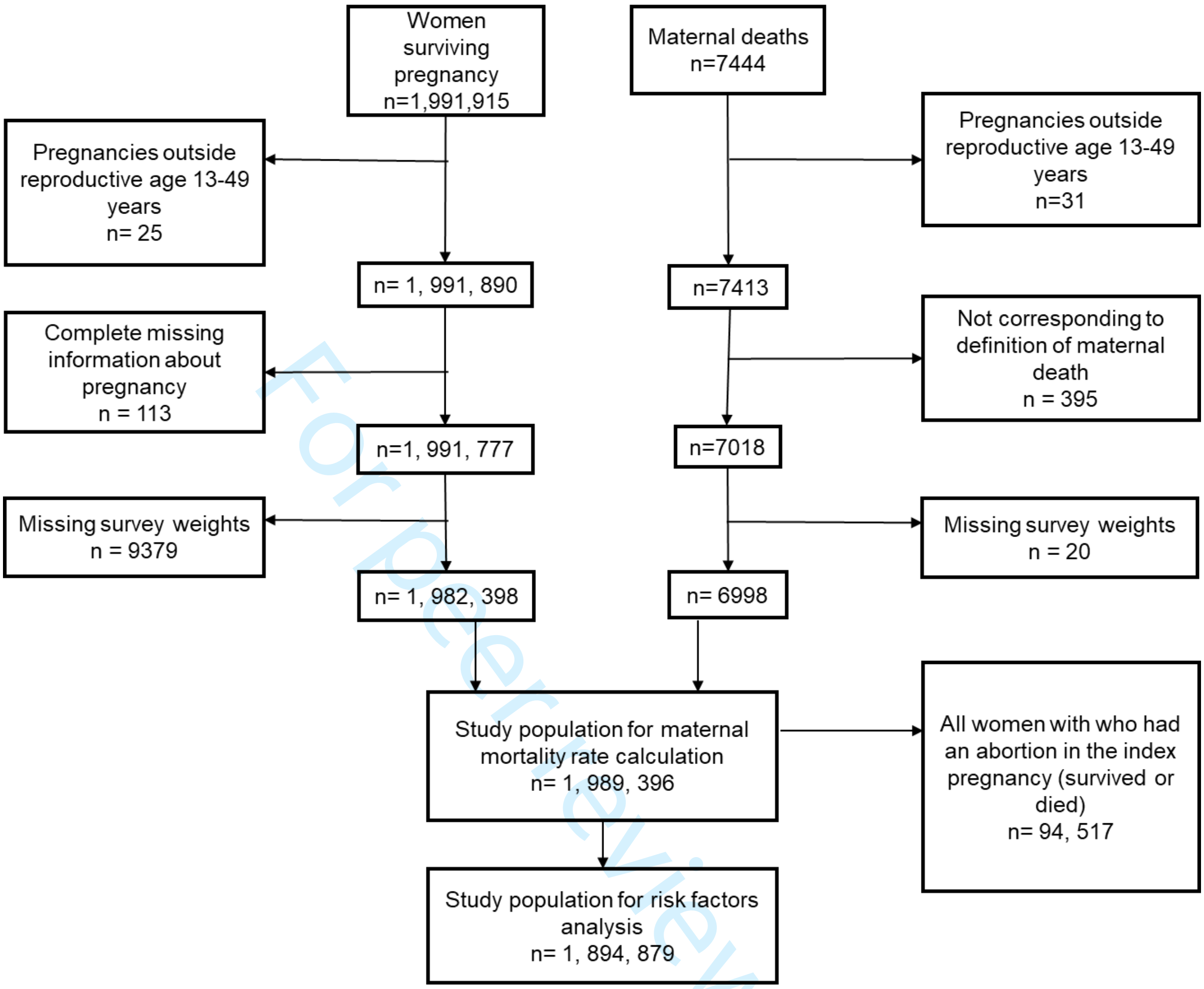

For peer review only

Figure S2: Theoretical framework for maternal mortality in India

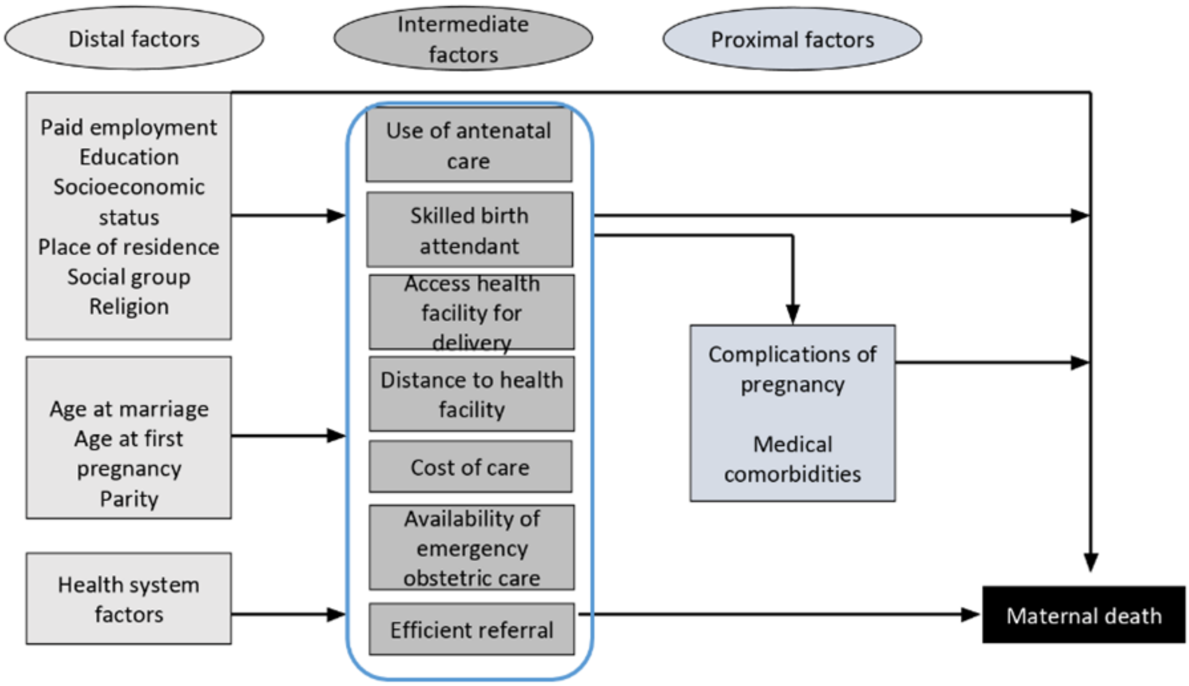

For peer review only

Figure S3: Odds ratios of maternal mortality according to maternal age with different levels of adjustment

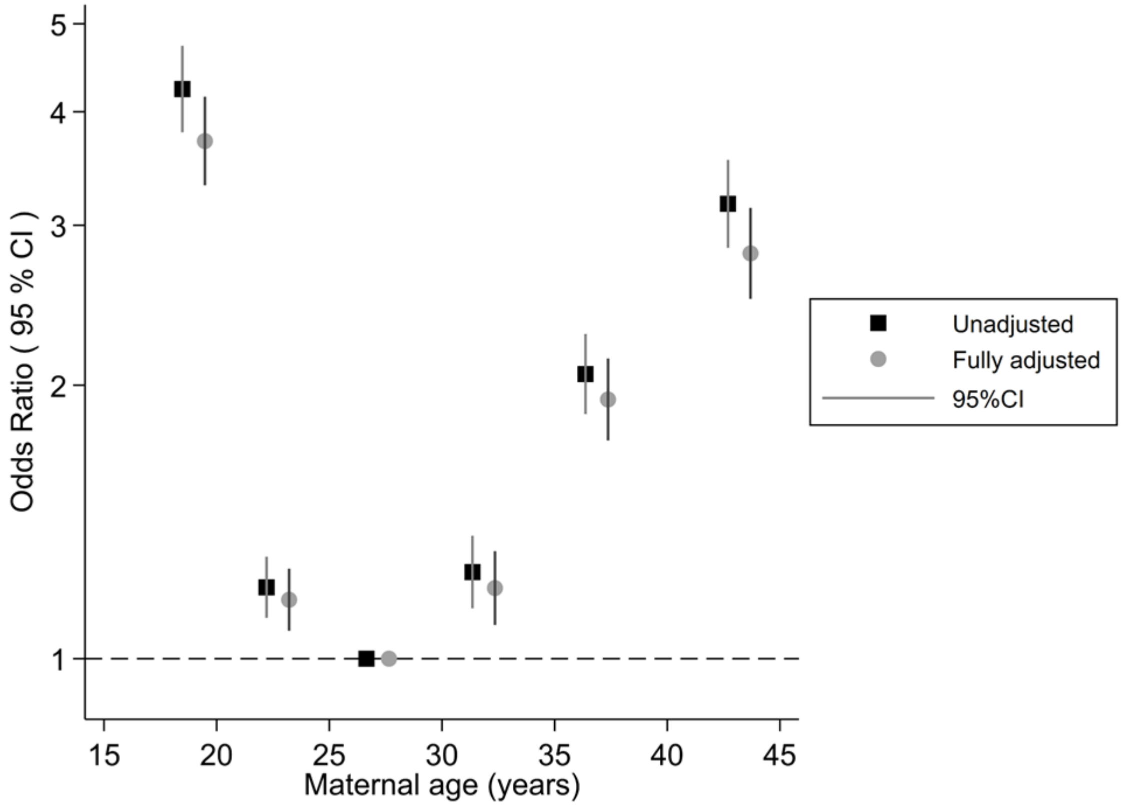

Note: Analyses were performed using logistic regression with adjustment for clustering and survey design. Odds ratios are shown with 95% confidence intervals. The fully adjusted model is fitted for all distal, intermediate and proximal factors.

Figure S4: Odds ratios of maternal death in rural vs. urban women with sequential adjustment

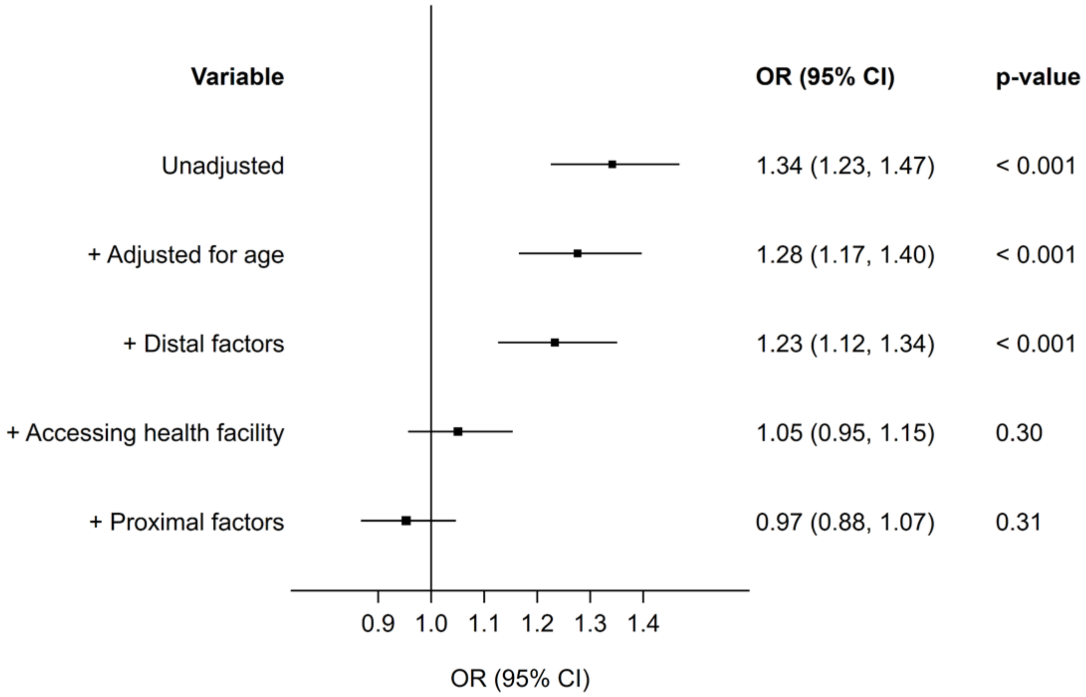

Note : Analyses were performed using logistic regression with adjustment for clustering and survey design. Size of data markers are proportional to the inverse of the variance of the odds ratio.

For peer review only - http://bmjopen.bmj.com/site/about/guidelines.xhtml

Figure S5: Odds ratio of maternal death in poorest vs. wealthiest women with sequential adjustment

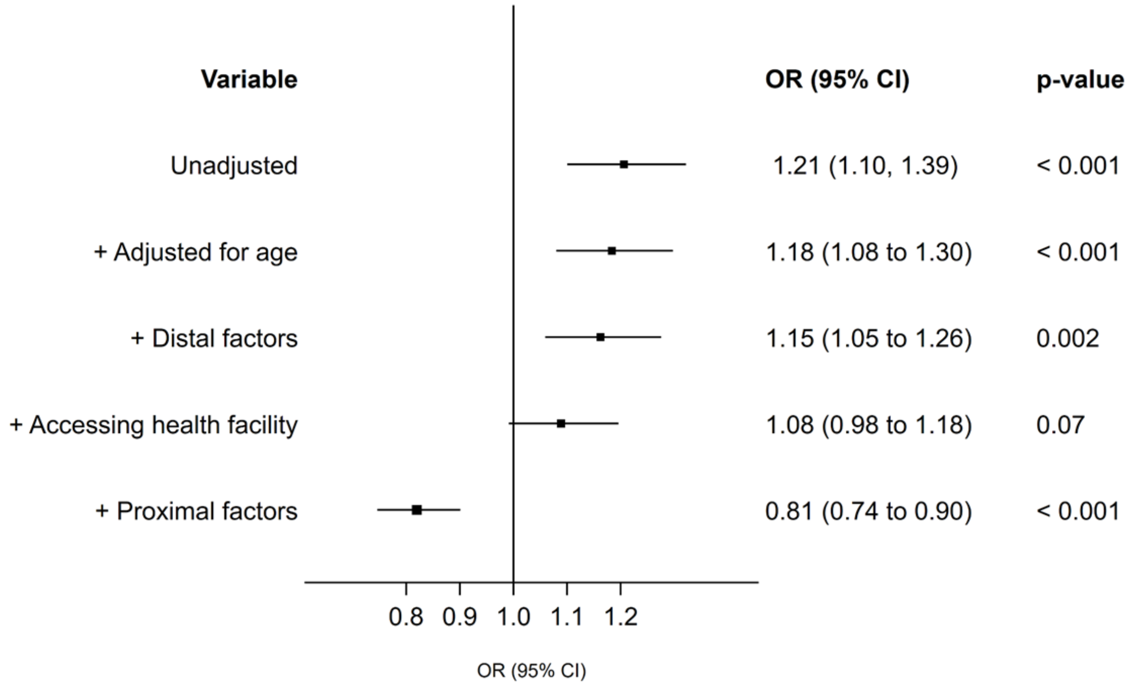

Note : Analyses were performed using logistic regression with adjustment for clustering and survey design. Size of data markers are proportional to the inverse of the variance of the odds ratio.

STROBE Statement—Checklist of items that should be included in reports of ***cross-sectional studies***

	Item No	Recommendation	Page No
**Title and abstract**	1	(*a*) Indicate the study's design with a commonly used term in the title or the abstract	1,2
		(*b*) Provide in the abstract an informative and balanced summary of what was done and what was found	2
**Introduction**			
Background/rationale	2	Explain the scientific background and rationale for the investigation being reported	4
Objectives	3	State specific objectives, including any prespecified hypotheses	4
**Methods**			
Study design	4	Present key elements of study design early in the paper	4
Setting	5	Describe the setting, locations, and relevant dates, including periods of recruitment, exposure, follow-up, and data collection	4,5
Participants	6	(*a*) Give the eligibility criteria, and the sources and methods of selection of participants	5
Variables	7	Clearly define all outcomes, exposures, predictors, potential confounders, and effect modifiers. Give diagnostic criteria, if applicable	4,5
Data sources/ measurement	8*	For each variable of interest, give sources of data and details of methods of assessment (measurement). Describe comparability of assessment methods if there is more than one group	5,6,7
Bias	9	Describe any efforts to address potential sources of bias	6,7
Study size	10	Explain how the study size was arrived at	5, Figure S1
Quantitative variables	11	Explain how quantitative variables were handled in the analyses. If applicable, describe which groupings were chosen and why	6,7, Table-1
Statistical methods	12	(*a*) Describe all statistical methods, including those used to control for confounding	6,7
		(*b*) Describe any methods used to examine subgroups and interactions	6,7
		(*c*) Explain how missing data were addressed	6,7
		(*d*) If applicable, describe analytical methods taking account of sampling strategy	6,7
		(*e*) Describe any sensitivity analyses	6,7
**Results**			
Participants	13*	(a) Report numbers of individuals at each stage of study—eg numbers potentially eligible, examined for eligibility, confirmed eligible, included in the study, completing follow-up, and analysed	5 and Figure-S1
		(b) Give reasons for non-participation at each stage	5 and Figure-S1
		(c) Consider use of a flow diagram	Figure-S1
Descriptive data	14*	(a) Give characteristics of study participants (eg demographic, clinical, social) and information on exposures and potential confounders	8,9, Table 1

For peer review only

		(b) Indicate number of participants with missing data for each variable of interest	Table 1
Outcome data	15*	Report numbers of outcome events or summary measures	5, Figure S1
Main results	16	(*a*) Give unadjusted estimates and, if applicable, confounder-adjusted estimates and their precision (eg, 95% confidence interval). Make clear which confounders were adjusted for and why they were included	Table-2
		(*b*) Report category boundaries when continuous variables were categorized	Table1, 2
		(*c*) If relevant, consider translating estimates of relative risk into absolute risk for a meaningful time period	Not applicable
Other analyses	17	Report other analyses done—eg analyses of subgroups and interactions, and sensitivity analyses	13, Figure 3
**Discussion**			
Key results	18	Summarise key results with reference to study objectives	13, 14
Limitations	19	Discuss limitations of the study, taking into account sources of potential bias or imprecision. Discuss both direction and magnitude of any potential bias	16,17
Interpretation	20	Give a cautious overall interpretation of results considering objectives, limitations, multiplicity of analyses, results from similar studies, and other relevant evidence	14,15,16,17
Generalisability	21	Discuss the generalisability (external validity) of the study results	15,16,17
**Other information**			
Funding	22	Give the source of funding and the role of the funders for the present study and, if applicable, for the original study on which the present article is based	19

*Give information separately for exposed and unexposed groups.

Note: An Explanation and Elaboration article discusses each checklist item and gives methodological background and published examples of transparent reporting. The STROBE checklist is best used in conjunction with this article (freely available on the Web sites of PLoS Medicine at http://www.plosmedicine.org/, Annals of Internal Medicine at http://www.annals.org/, and Epidemiology at http://www.epidem.com/). Information on the STROBE Initiative is available at www.strobe-statement.org.

For peer review only
